# Dentin sialoprotein facilitates dental mesenchymal cell differentiation and dentin formation

**DOI:** 10.1038/s41598-017-00339-w

**Published:** 2017-03-22

**Authors:** Wentong Li, Lei Chen, Zhuo Chen, Lian Wu, Junsheng Feng, Feng Wang, Lisa Shoff, Xin Li, Kevin J. Donly, Mary MacDougall, Shuo Chen

**Affiliations:** 1 Department of Developmental Dentistry, the University of Texas Health Science Center at San Antonio, San Antonio, Texas 78229-3700 United States; 2 Department of Pathology, Weifang Medical University, Weifang, Shandong Province 261053 China; 3 Department of Surgery, the First Affiliated Hospital, Fujian Medical University, Fuzhou, Fujian 350108 China; 4 Department of Oral/Maxillofacial Surgery, University of Alabama at Birmingham School of Dentistry, Birmingham, Alabama 35294-0007 United States

## Abstract

Dentin sialoprotein (DSP) is a dentin extracellular matrix protein. It is involved in dental mesenchymal cell lineages and dentin formation through regulation of its target gene expression. DSP mutations cause dentin genetic diseases. However, mechanisms of DSP in controlling dental mesenchymal cell differentiation are unknown. Using DSP as bait, we screened a protein library from mouse odontoblastic cells and found that DSP is a ligand and binds to cell surface receptor, occludin. Further study identified that the C-terminal DSP domain^aa 363–458^ interacts with the occludin extracellular loop 2^aa 194–241^. The C-terminal DSP domain induced phosphorylation of occludin Ser^490^ and focal adhesion kinase (FAK) Ser^722^ and Tyr^576^. Coexpression of DSP, occludin and FAK was detected in dental mesenchymal cells during tooth development. Occludin physically interacts with FAK, and occludin and FAK phosphorylation can be blocked by DSP and occludin antibodies. This DSP domain facilitates dental mesenchymal cell differentiation and mineralization. Furthermore, transplantation and pulp-capping procedures revealed that this DSP domain induces endogenous dental pulp mesenchymal cell proliferation, differentiation and migration, while stimulating blood vessel proliferation. This study elucidates the mechanism of DSP in dental mesenchymal lineages and implies that DSP may serve as a therapeutic agent for dentin-pulp complex regeneration in dental caries.

## Introduction

Craniofacial skeleton is original from neural crest-derived mesenchymal cells^1^. These cells proliferate and differentiate into odontoblasts and osteoblasts as well as finally build dynamic mineralized tissues such as bone and dentin. In this process, cell proliferation and differentiation are tightly controlled by spatiotemporal cell-cell interaction and extracellular matrix (ECM) to ensure that the tissue attains specific size, shape, structure, and function.

ECM often provides specific microenvironments (niches) necessary for controlling morphology, cell fate specification, cell migration and tissue repair^2^. Degradation or activation of ECM proteins by proteolysis during growth, morphology and tissue repair can mediate rapid and irreversible responses to changes in the cellular niches and cell homeostasis^3^. ECM in bone and dentin mainly comprises a number of collagenous and non-collagenous proteins (NCPs). Among the NCPs, a family of small integrin-binding ligand N-linked glycoproteins (SIBLINGs) comprises bone sialoprotein (BSP), dentin matrix protein 1 (DMP1) and dentin sialophosphoprotein (DSPP), matrix extracellular phosphoglycoprotein (MEPE) and osteopontin (OPN). These SIBLING genes are highly expressed in mineralizing tissues related to tooth and bone development and believed to be responsible for initiating and modulating cell differentiation and mineralization processes via matrix-cell interaction. For instance, an Arg-Gly-Asp (RGD) triple peptide within several NCPs regulates intracellular signal pathways via cell membrane receptors such as integrin^4^. Despite their common origin, dentin and bone are dramatically different from their morphologies and physical functions. One of great differences is DSPP in the two tissues. Spatial and temporal expression of DSPP is largely restricted to odontoblasts and dentin^5, 6^. Expression of DSPP in odontoblasts and dentin is approximately 400 fold higher than that of osteoblasts and bone^7^. Although DSPP is transcribed from a single gene^8, 9^, full length of DSPP protein has scarcely been isolated from cells or tissues^10, 11^, whereas its cleavage products, dentin sialoprotein (DSP) and dentin phosphoprotein (DPP), are most abundant NCPs in odontoblasts and dentin^12^. DSP is further processed into small molecular fragments^11, 13–15^. Cleaved DSP fragments segregate into specific compartments within odontoblasts and dentin^14, 16^.

DSP and DPP play unique biological functions during tooth development^17, 18^. Mutations of either DSP or DPP domain in humans caused dentinogenesis imperfecta (DGI) type II (DGI-II, OMIM #125490) and type III (DGI-III, OMIM 125500) and dentin dysplasia (DD) type II (DD-II, OMIM 125420)^19–21^, the most common dentin genetic diseases. Mouse DSPP knock-out exhibited similar phenotype to that of DSPP gene mutations in human^22^. DPP contains an RGD domain, acting as a ligand, and binds to integrin as well as triggers intracellular signals via DPP-RGD/integrin-αvβ3 interactions^23, 24^. By contrast, DSP lacks a RGD domain^9^, and many DSPP gene mutations occur in DSP region^19, 20, 25^. DSP and peptides derived from DSP are able to regulate gene expression and protein phosphorylation as well as induce dental primary/stem cell differentiation^9, 16, 26^. Recently, we have identified that 36 amino acids of DSP domain^aa 183–219^ bind to integrin β6 and the DSP-integrin β6 complex stimulated phosphorylation of Smad1/5/8 proteins through p38 and Erk 1/2 protein kinases. The phosphorylated Smad1/5/8 proteins were translocalized into nuclei and bind to DSPP gene promoter, activating expression of DSPP and DMP1 genes and inducing dental mesenchymal cell differentiation and biomineralization^9^. However, the molecular mechanisms of DSP controlling gene expression and cell differentiation have not been completely understood.

Occludin (Ocln) is an integral membrane protein associated with the tight junctions (TJs) of cells and mainly comprises four transmembrane domains, NH_2_- and COOH-terminal cytoplasmic regions and two extracellular loops^27, 28^. The COOH-terminal domain is rich in serine, threonine and tyrosine residues, which are frequently phosphorylated by various protein kinases^29^. The extracellular loops of Ocln interact with a variety of cellular signaling molecules and are dynamically involved in intracellular signal transductions including protein phosphorylation/dephosphorylation and ion flux^28, 30, 31^. The cytoplasmic tail of Ocln is necessary for binding to its partners^32^. Ocln mutations in humans are involved in the pathogenesis of malformations of cortical development with band-like brain calcification and chronic kidney dysfunction^15, 33–35^. *Ocln* deficient mice developed deafness with dislocalization of tricellulin in cochlea^36^ and displayed other signs of pathological disorders such as growth retardation, dysfunction of the salivary gland, calcification in the brain and thinning of the compact bone^37^. Although Ocln is one of the TJ proteins, unexpectedly, *Ocln* knock-out mice exhibited the normal TJ strand formation, and visceral endoderm cells originating from *Ocln*-deficient embryonic stem cells have well-developed networks of tight-junction strands^37, 38^. It suggested that Ocln is also important for other biological functions. However, whether the signaling pathways by which DSP regulates intracellular activity via Ocln is not clearly understood.

Focal adhesion kinase (FAK or Ptk2) is the non-receptor bound tyrosine kinase and is involved in mediating both integrin and Ocln signaling for regulating intracellular transduction with the ECM on the outside of the cells^39, 40^. Phosphorylation events occurring within focal adhesions influence numerous processes including cell adhesion, shape, motility as well as cell differentiation and tissue development^41, 42^. FAK knockout studies showed an early embryonic lethal phenotype with extensive mesodermal deficiency and Ptk2^−/−^ embryonic fibroblasts from these mice exhibited profound defects in migration^43^.

Our hypothesis is that DSP regulates intracellular signal transductions and dental mesenchymal cell differentiations via matrix-cell surface interaction. Here, we found that DSP as a ligand is capable of binding to its cell surface receptor, Ocln. Further researches revealed that the COOH-terminal DSP domain interacts with the extracellular loop 2 of Ocln. This peptide phosphorylates Ocln and FAK. Immunohistochemistry showed that the spatial-temporal distribution of DSP, Ocln and FAK is co-localized in odontoblasts during dentinogenesis. The DSP domain was capable of inducing differentiation and mineralization of dental pulp stem cells. Furthermore, this peptide induced endogenous mouse dental pulp cell proliferation and differentiation and enhanced biomineralization when this DSP peptide was implanted into mouse dental pulp chambers.

## Results

### DSP as a ligand binds to its cell surface receptor, Ocln

**Figure 1:**
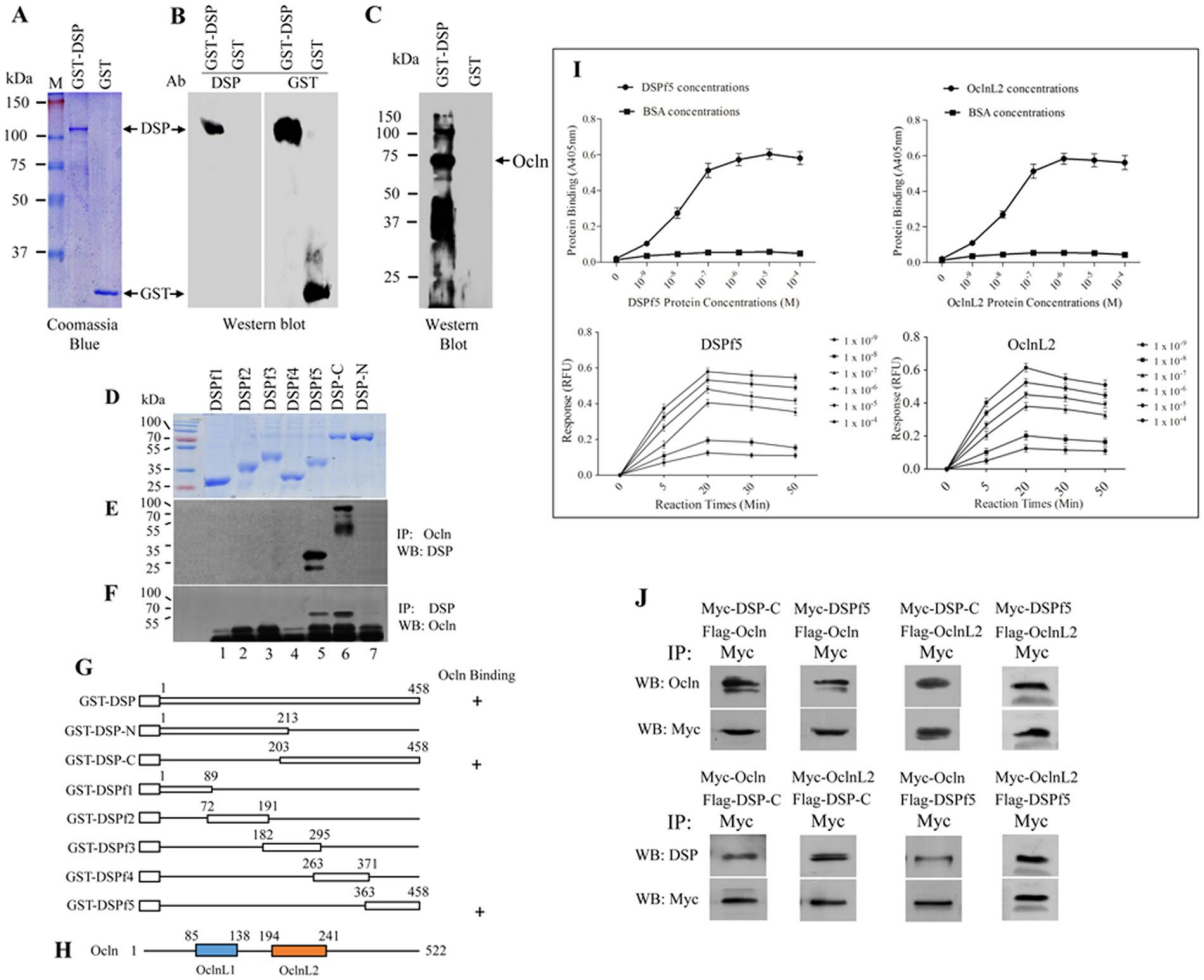
DSP interacts with occludin. Recombinant DSP 1–458, 1–213, 203–458, 1–89, 72–191, 182–295, 263–371, 363–458 were expressed in *Escherichia coli* BL21 and purified according to the manufacturer's instruction as described by “Materials and methods”. The purified DSP fusion proteins were confirmed by Coomassie blue staining (**A,D**) and Western blot assays using either an anti-GST or anti-DSP antibody (**B**). Interaction between DSP polypeptides and Ocln by GST pull down was detected by Western blot using anti-DSP and anti-Ocln antibodies (**C,E,F**). Arrow shows Ocln band (**C**). Mixture of different fragments of DSP and Ocln fusion proteins was pulled down by Ocln antibody and interaction of DSP with Ocln was detected using DSP antibody (**E**) and vice versa **(F)**. The GST-DSP fusion protein constructs and the localization of the DSP protein stretch that contains Ocln-binding domain are illustrated (**G**). The DSP number starts at the translational start site of DSPP (Met) as No. 1. (**H**) Schematic representation of mouse Ocln protein structure from the initiation of the translation start site 1 to the end 522. OclnL1 and OclnL2 indicate the extracellular loop 1 (aa 85–183) and loop 2 (aa 194–241). **(I)** Either recombinant OclnL2 protein or BSA as control was coated in 96-well plates. Serial diluted biotinylated DSPf5 was added to the wells and incubated with the unlabeled recombinant OclnL2 protein or BSA, respectively. Bound DSPf5 was detected using AP-conjugated streptavidin and 1 mg/ml PNPP as substrate at 405 nm using a microplate reader. Binding affinity of different concentrations of DSPf5 to its substrate at the given time periods were calculated. Using the same method, binding of the biotinylated OclnL2 to the unlabeled DSPf5 was examined. Data point represent the mean ± S.D. (n = 3). **(J)** For *in vivo* studies, different fragments of DSP and Ocln cDNAs were subcloned into a CMV mammalian expression plasmid tagged with Flag or Myc peptides, respectively. Myc-Ocln and Flag-DSP as well as Myc-DSP and Flag-Ocln expression vectors were transfected into HEK-293 cells, respectively. After 48 h transfection, the proteins were harvested and protein-protein interactions were immunoprecipitated using Myc antibody. Protein-protein interaction was detected by Western blotting using anti-Myc, anti-DSP or anti-Ocln antibody.

To identify whether DSP interacts with other proteins, we generated GST-DSP fusion protein (Fig. 1A,B), and cell lysis was isolated from mouse odontoblast-like cells. The DSP fusion protein was used as bait to screen the protein library. This result showed that four proteins among 110 candidates interact with DSP, including integrin β6, CD105 (endoglin), collagen IV and Ocln (Fig. 1C, Table 1. Supplementary Table 1). To further identify which domain of DSP binds to Ocln, we generated different DSP and *Ocln* gene constructs for protein-protein interactions. These results showed that the COOH-terminal DSP domain^aa 363–458^ (DSPf5) interacts with the extracellular loop 2 of Ocln^aa 194–241^ (OclnL2) (Fig. 1E–H). To further verify specific and saturable interactions and to evaluate the binding affinity between DSPf5 and OclnL2, we used a concentration range of DSPf5 and OclnL2 as analyzed by biotin-labeled proteins. These results manifested that the binding of DSPf5 to OclnL2 was dose- and time-dependent manners (Fig. 1I). For *in vivo* study, mammalian expression of DSP and *Ocln* genes was co-transfected into mammalian human embryonic kidney (HEK) 293 cells and coimmunoprecipitation assay showed that DSP binds to Ocln, and binding of DSP to Ocln was narrow to DSPf5 and OclnL2 (Fig. 1J), whereas the DSP-Ocln interaction could be blocked by the DSP and Ocln antibodies, but not IgG as control (Supplementary Fig. 1). Using the gene runner computer software analysis of amino acids of the OclnL2 and DSPf5 reveals that these regions across many species are highly homologous (Supplementary Fig. 2A,B). This result indicates that DSP acts as a ligand and binds to its cell surface receptor, Ocln.

**Table 1:** Expression, signaling pathway and function of the four proteins bound by dentin sialoprotein.

gene	Expression feature	Signaling pathway	Relations to human disorder	Interaction molecules
Collagen IV	The major structural component of glomerular basement membranes	Interleukin-3, 5 and GM-CSF signaling and Pathways in cancer	Alport Syndrome, Autosomal Recessive and Alport Syndrome, Autosomal Dominant	Forming a chicken-wire meshwork together with laminins, proteoglycans and entactin/nidogen.
Endoglin	Expressed in endothelial cells, smooth muscle cells, vascular smooth muscle cells	TGF-β Receptor Signaling Pathway	Telangiectasia, Hereditary Hemorrhagic, Type 1 and Hereditary Hemorrhagic Telangiectasia, Tumor angiogenesis, tumor growth and metastasis	Interacted with zyxin, ZRP-1, β-arrestin,Tctex2β, LK1, ALK5, TGFβ II, and GIPC
Integrin β6	Proliferating epithelia, esp. lung and mammary gland	ERK, Rho Family, GTPases, MAPK, FAK1 Signaling	Amelogenesis Imperfecta, Type Ih and Hypocalcified Amelogenesis Imperfecta, tumor growth and metastasis	Fibronectin; TGF-β I, dentin sialoprotein
Occludin	Widely distributed in the epithelial cells and endothelial cells of brain, liver, lung, kidney	VCAM-1/CD106 Signaling Pathways and Blood-Brain Barrier Pathway	Band-Like Calcification With Simplified Gyration and Polymicrogyria	Interact with ZO-1, ZO-2, ZO-3, Claudin and other proteins to form tight junction

### Expression of DSP and Ocln during mouse tooth formation

**Figure 2:**
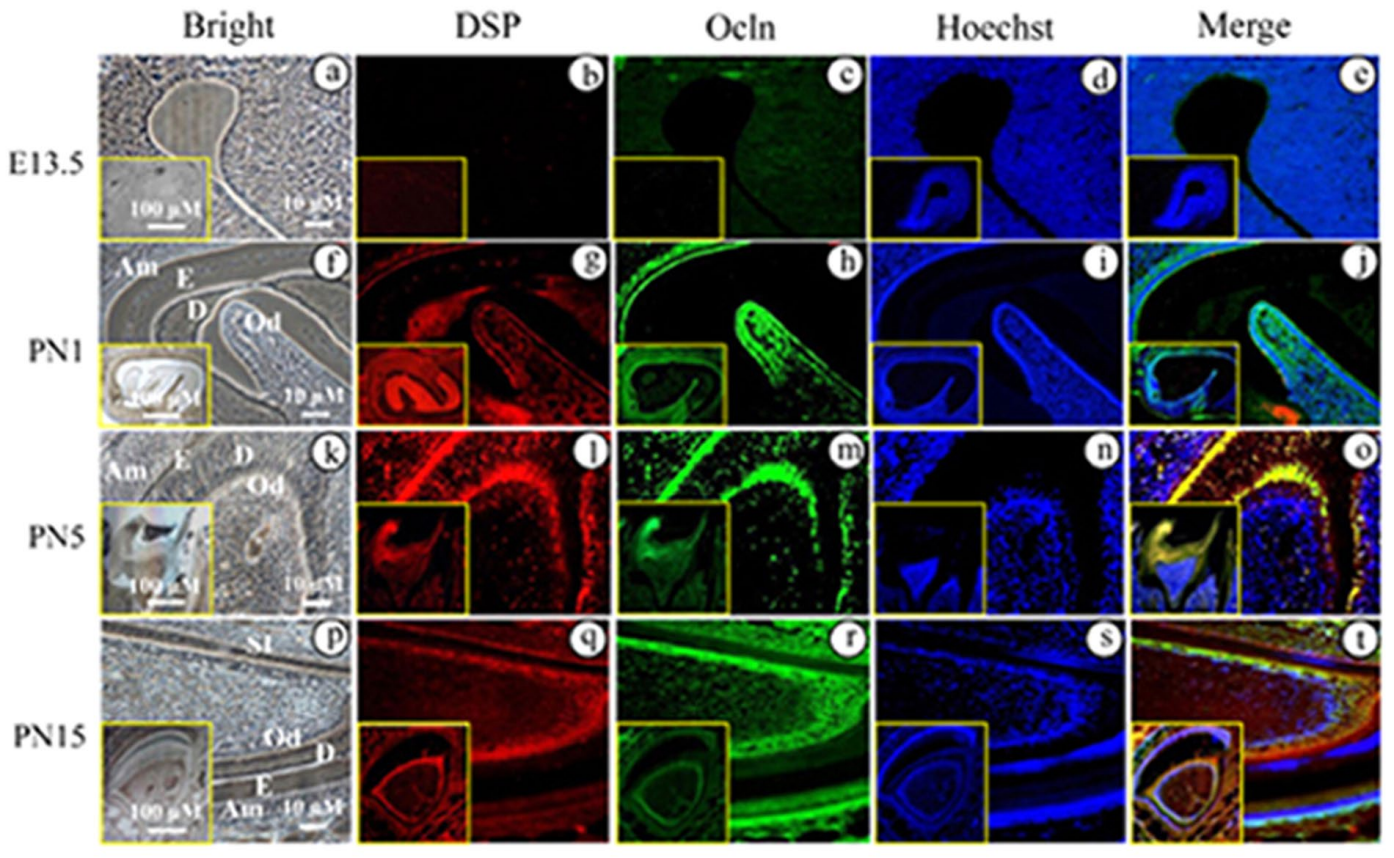
Expression of dentin sialoprotein and occludin in developing mouse teeth. At embryonic day (E) 13.5, DSP and Ocln expressions were not detected in tooth tissues (**b,c**) using double fluorescent immunohistochemistry, but at postnatal day (PN) 1, DSP expression (red) was observed in odontoblasts, ameloblasts and dental pulp cells (**g**), Ocln expression (green) was detected in these areas overlapped with DSP (**h**). At PN5 and PN15, expression of DSP and Ocln was apparently seen in odontoblasts and ameloblasts (**l,m,q,r**). However, Ocln expression was relatively wider than that of DSP. (**a,f,k** and **p**) show bright images. The cells were stained with Hoechst for nuclei (**d,i,n,s**). Images were merged (**e,j,o,t**). (**a**–**t**) show higher magnifications from the yellow boxes.

To determine whether the two proteins are coexpressed in tooth tissues, their distribution profiles during developing mouse teeth were examined using double labeling immunohistochemistry. At embryonic day (E) 13.5, DSP and Ocln expressions were not detected in craniofacial tissues (Fig. 2b,c). At postnatal day 1 (PN1), DSP was expressed in odontoblasts and weakly in ameloblasts (Fig. 2g). At PN5, DSP expression was strongly observed in odontoblasts and dentin and moderately in ameloblasts (Fig. 2l). Similar to PN5, DSP distribution exhibited the same expression pattern at PN15 in mouse teeth (Fig. 2q). Ocln signal coincided with DSP expression in odontoblasts (Fig. 2h,m,r), but Ocln expression pattern was also seen in stratum intermedium (SI). Although Ocln is an integral membrane protein, the NH_2_-and COOH domains of Ocln are localized in the cytoplasm^22^. Therefore, Ocln expression was observed in the cytoplasm besides the cell membrane. No signal was detected in the negative control tissue sections, where normal IgG was used instead of the primary antibodies of DSP and Ocln (Supplementary Fig. 3b,c).

### DSP induces phosphorylation of Ocln and FAK and two protein expressions in odontoblasts

**Figure 3:**
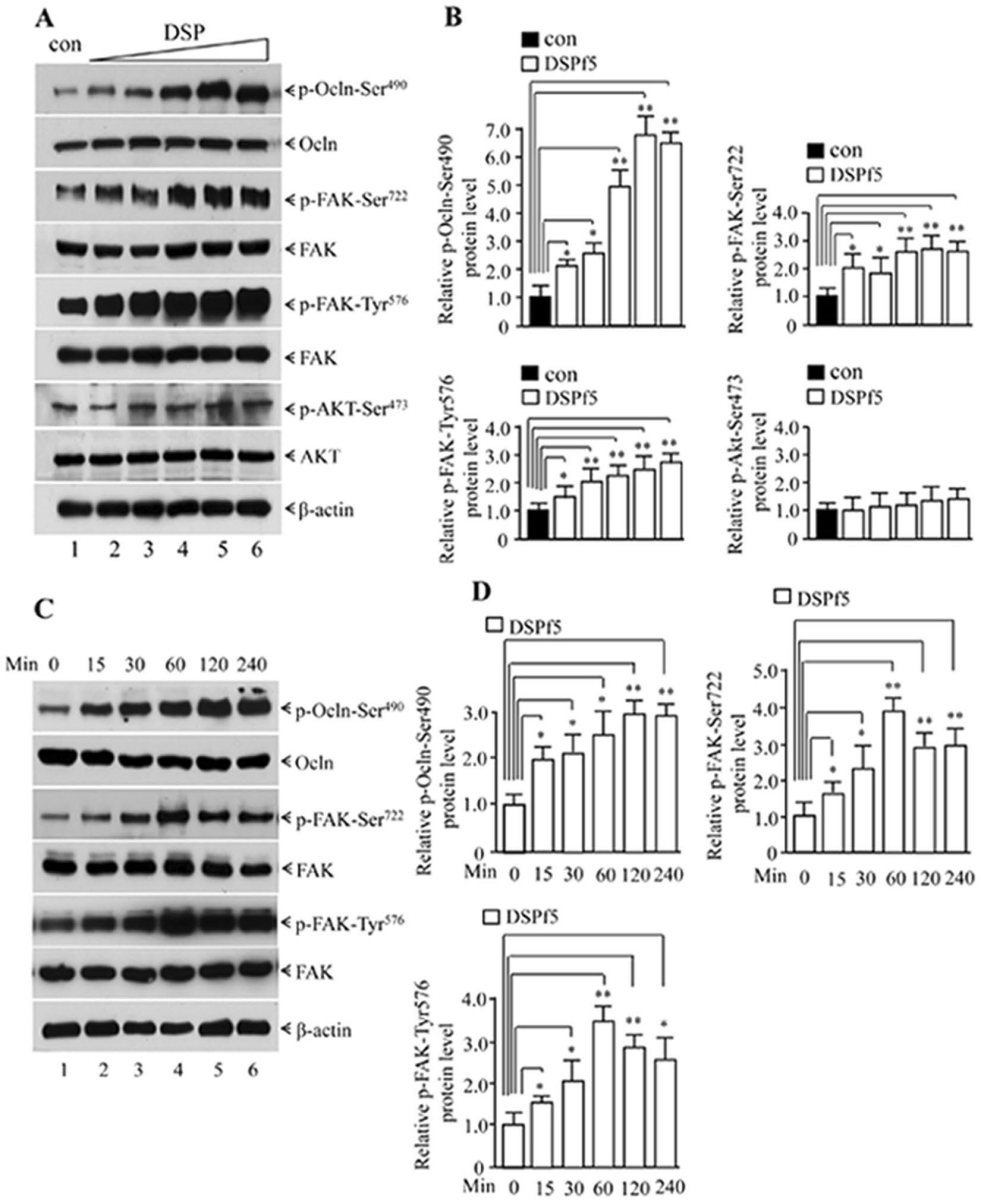
Effect of DSPf5 on occludin and FAK phosphorylation. (**A**) The mouse dental papilla mesenchymal cells were treated with or wither DSPf5 of 2 μg/ml (lane 2), 4 μg/ml (lane 3), 8 μg/ml (lane 4), 16 μg/ml (lane 5) and 24 μg/ml (lane 6) for 1 h at 37 °C. After cell harvest, protein expression was detected by Western blot assay using antibodies specific to p-Ocln-Ser^490^, Ocln, p-FAK-Ser^722^, p-FAK-Tyr^576^, FAK, p-AKT-Ser^473^, AKT and β-actin. (**B**) Protein expression levels were quantitated using imageJ software. Expression of p-Ocln-Ser^490^, p-FAK-Ser^722^, p-FAK-Tyr^576^ and p-AKT-Ser^473^ was normalized to Ocln, FAK and AKT, respectively. Protein on the control was considered as a one-fold increase. The expression level of proteins treated with DSPf5 was divided by protein level on the control group. The results showed the mean ± S.D. (n = 3). *p < 0.05; **p < 0.01. (**C**) The cells were treated with DSPf5 (8 μg/ml) in DMEM medium for 0. 15, 30, 60, 120 and 240 min. Protein expression was detected by Western blot analysis using antibodies specific to p-Ocln-Ser^490^, Ocln, p-FAK-Ser^722^, p-FAK-Tyr^576^, FAK, and β-actin, respectively. (**D**). Protein expression levels were quantitated using image J software. Expression of p-Ocln-Ser^490^, p-FAK-Ser^722^ and p-FAK-Tyr^576^ was normalized to Ocln and FAK, respectively. Protein treated with DSPf5 at 0 min as control group was considered as a one-fold increase. The expression level of proteins treated with DSPf5 at different time periods was divided by protein level on the control group.

To elucidate whether DSP regulates modification of Ocln and its partner activity, mouse dental papilla mesenchymal cells were treated with DSPf5 protein. We found that DSPf5 phosphorylates Ocln at Ser^490^ at a dose-dependent manner (Fig. 3A,B). As several genes are Ocln partners^29^, we further investigated whether DSPf5 also regulates Ocln partner activity. Interestingly, we found that DSPf5 also induces FAK phosphorylation at Ser^722^ and Tyr^576^, but no effect on FAK at Tyr^397^ (data not shown) and Akt at Ser^473^ (Fig. 3A,B). Maximal induction of protein phosphorylation of Ocln-Ser^490^ and FAK-Ser^722^, FAK-Tyr^675^ by DSPf5 was at 2 h, 1 h and 1 h, respectively (Fig. 3C,D).

**Figure 4:**
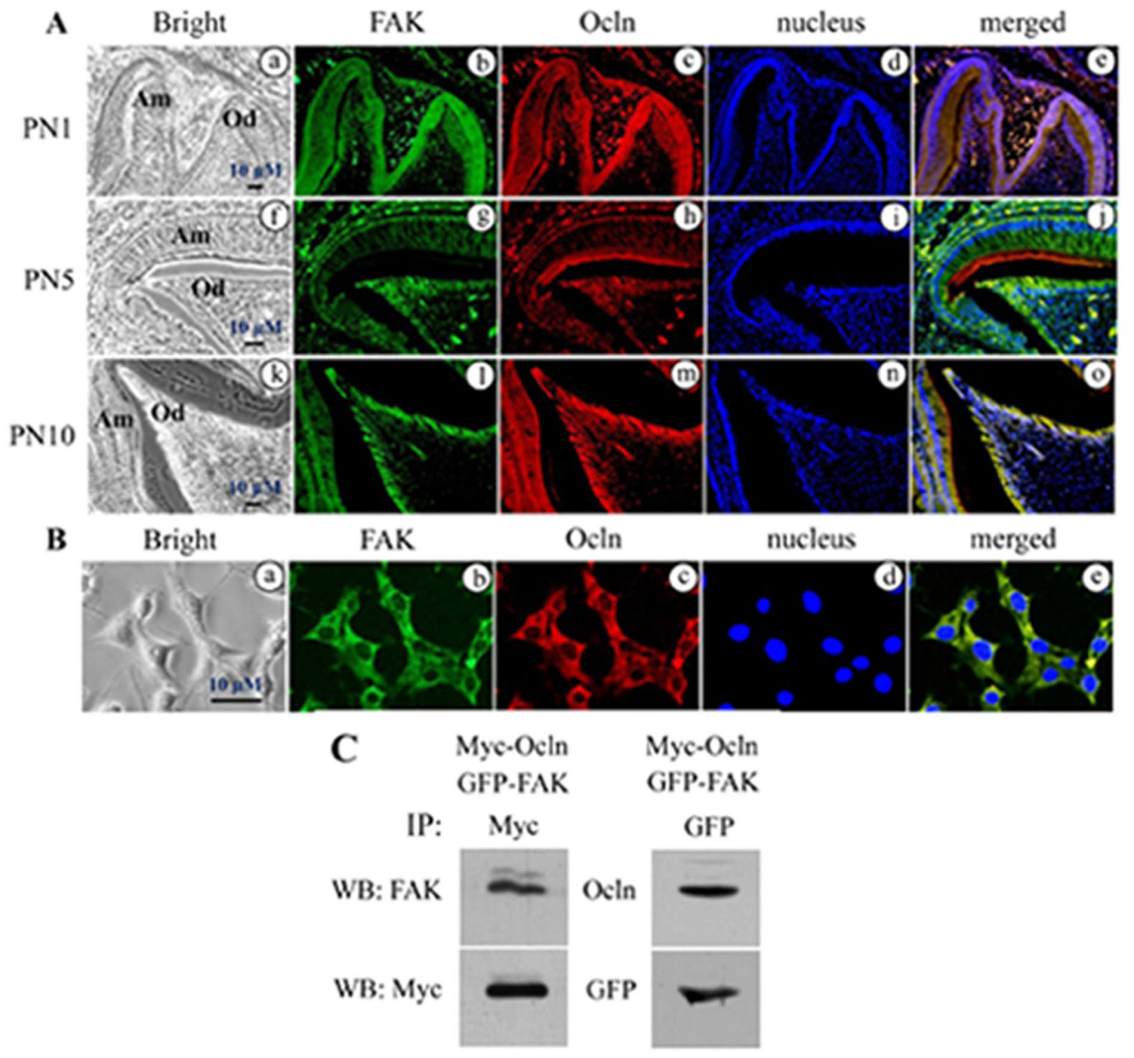
Expression of occludin and FAK in odontoblasts during tooth development. (**A**) Fluorescent immunohistochemistry showed that at PN1, 5, 10 during mouse tooth development, Ocln (red) and FAK (green) were co-expressed in odontoblasts by the double immunofluorescent histochemistry using antibodies specific to Ocln and FAK (**b,c,g,h,l,m**). (**a**, **f** and **k**) show bright images. The cells were stained with Hoechst for nuclei (**d,i,n**). Images were merged (**e,j,o**). Am, Ameloblasts; Od, Odontoblasts. (**B**) Ocln (red) and FAK (green) were expressed in iMDP-3 cells using double immunofluorescent assay (**b,c**). **a** shows bright image. Hoechst was used for nuclear staining (**d**). Image was merged (**e**). **(C)** Ocln interacts with FAK *in vivo*. Myc-*Ocln* and GFP-FAK mammalian expression vectors were cotransfected into HEK-293 cells. After 48 h transfection, the cells were harvested. Ocln and FAK were immune-precipitated using Myc or GFP antibody. Interaction of Ocln with FAK was detected by Western blot assay using Ocln and FAK antibodies.

To determine relationship between Ocln and FAK within odontoblasts during odontogenesis, expression of Ocln and FAK in odontoblasts was performed using double labeling fluorescent immunohistochemistry. Our study showed that expression of Ocln and FAK was observed in odontoblasts at PN1, 5, and 10 (Fig. 4A). No signals were detected in the control tissue sections when the antibodies of Ocln and FAK were replaced by normal IgG as negative control (Supplementary Fig. 4b,c). Also, expression of Ocln and FAK proteins was seen in mouse dental papilla mesenchymal cells (Fig. 4B) and HEK 293 cells (Supplementary Fig. 4g,h), but no immunostaining was seen in the control groups (Supplementary Fig. 4l,m,q,r). To further elucidate if Ocln interacts with FAK, mammalian expression vectors of *Ocln* and FAK genes were transfected into mammalian cells. Coimmunoprecipitation assay demonstrated that Ocln physically interacts with FAK *in vivo* (Fig. 4C).

### Effect of DSPf5 on phosphorylation of Ocln and FAK is disrupted by DSP and Ocln antibodies

**Figure 5:**
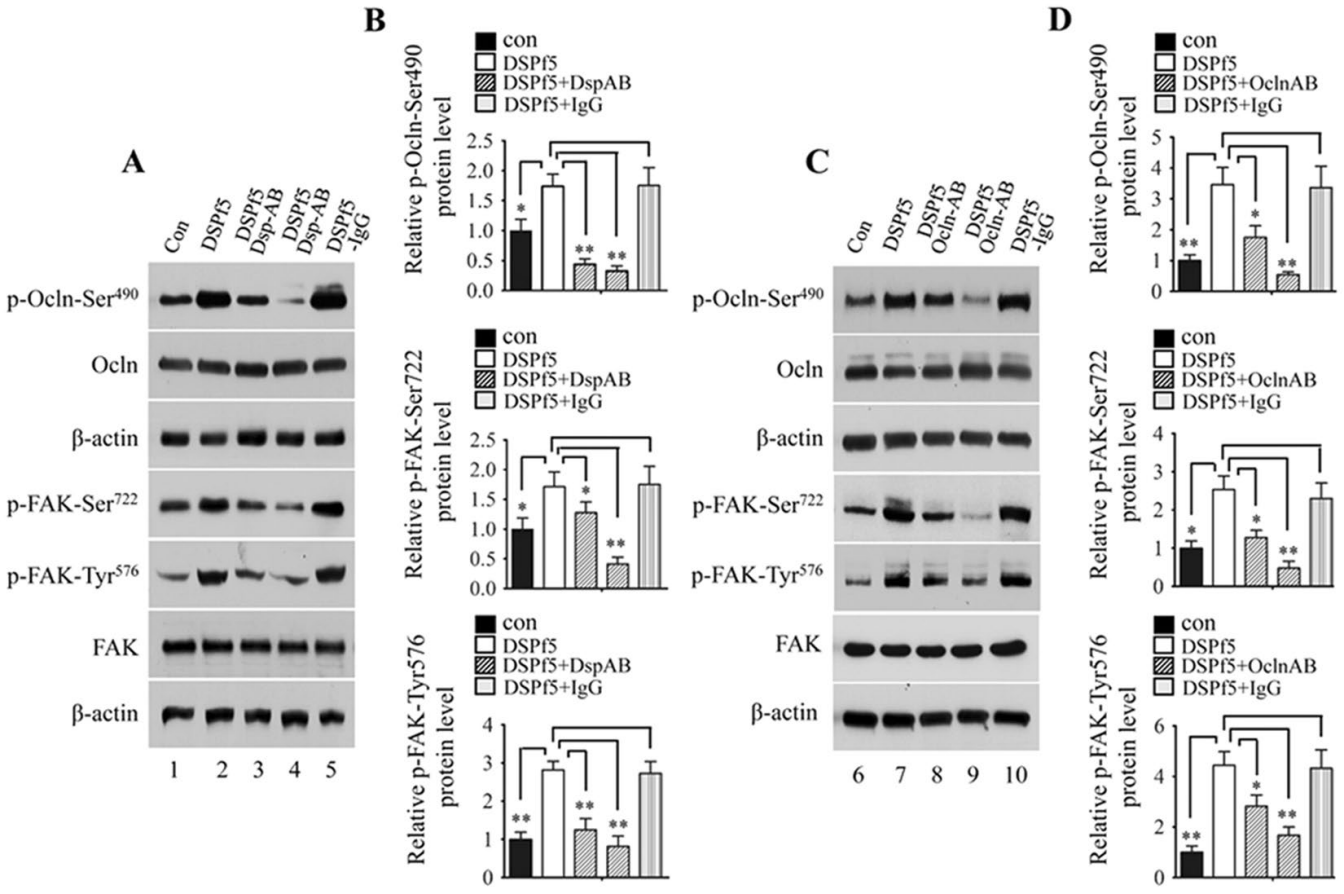
Effect of DSPf5 on occludin and FAK phosphorylation is blocked by DSP and occludin antibodies. (**A,C**) The mouse dental papilla mesenchymal cells were treated with or without DSPf5, or DSPf5 plus DSP antibody or DSPf5 plus Ocln antibody or DSPf5 with IgG as control for 1 h, respectively. Protein expression was detected by Western blotting using antibodies described above. Lanes, 1 and 6 as control without the DSPf5 induction; lanes 2 and 7 (8 μg/ml of DSPf5); lane 3 (8 μg/ml of DSPf5 plus 8 μg/ml of DSP antibody); lane 4 (8 μg/ml of DSPf5 plus 16 μg/ml of DSP antibody); lane 8 (8 μg/ml of the DSPf5 plus 8 μg/ml of Ocln antibody); lane 9 (8 μg/ml of DSPf5 plus 16 μg/ml of Ocln antibody); lanes 5 and 10 (8 μg/ml of DSPf5 plus 16 μg/ml of IgG). (**B,D**) Protein expression level was measured using imageJ software. Expression of p-Ocln-Ser490, p-FAK-Ser722 and p-FAK-Tyr576 was normalized to Ocln and FAK, respectively. Protein without treatment was considered as one-fold increase. The expression protein level of the treated groups was divided by protein level on the control group. The results are shown as the mean ± S.D. (n = 3). *p < 0.05; **p < 0.01.

**Figure 6:**
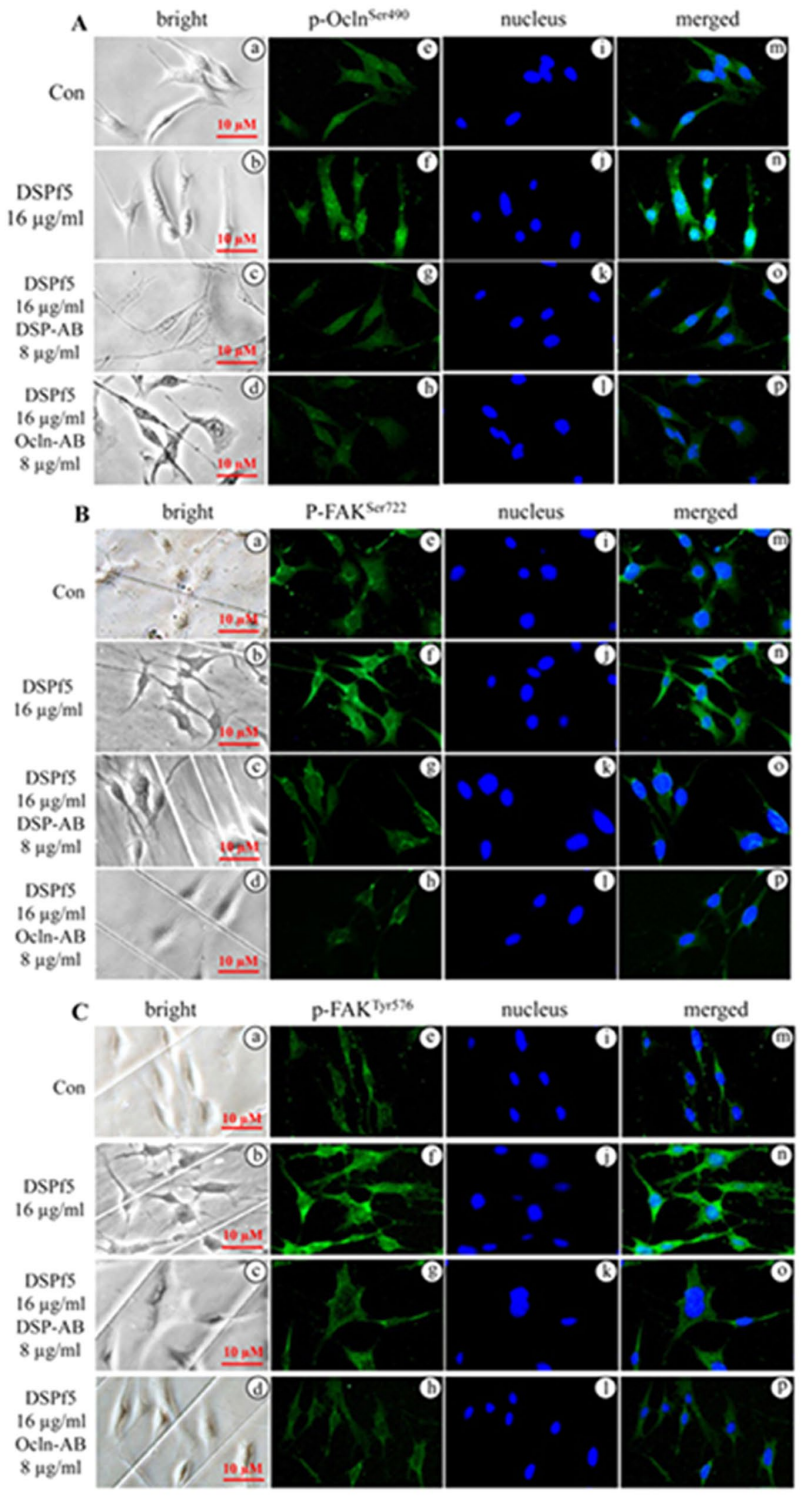
DSP and occludin antibodies block phosphorylation of occludin and FAK proteins mediated by DSPf5 in mouse dental papilla mesenchymal cells. (**A–C**) The cells were treated with or without DSPf5 or DSPf5 (16 μg/ml) plus the DSP (8 μg/ml) or Ocln (8 μg/ml) antibody for 1 h at 37 °C. The cells were fixed and immune-stained using p-Ocln-Ser^490^ (**A**), p-FAK-Ser^722^ (**B**) and pFAK-Tyr^576^ (**C**) antibodies, respectively. Data showed that effect of DSPf5 on Ocln and FAK phosphorylation was blocked by the DSP or Ocln antibody (e–h). (a–d) are bright images. The cells were stained with Hoechst for nuclei (i–l). Images were merged (m–p).

To further assess phosphorylation of Ocln and FAK mediated by DSP-Ocln signaling, we sought to block DSP signal pathway using DSP and Ocln antibodies. Mouse dental papilla mesenchymal cells were treated by DSPf5 with or without the DSP or Ocln antibody and the cells were harvested for immunoblot analysis. It was noted that the DSP and Ocln antibodies could block phosphorylation of Ocln at Ser^490^ and FAK at Ser^722^ and Tyr^576^ compared to the control group (Fig. 5A-D), but IgG as control had no effect on block of the two protein phosphorylations induced by DSPf5. Also, disruption of the two protein phosphorylation by the DSP and Ocln antibodies was dose-dependent. With increase of the DSP and Ocln antibodies, phosphorylation of p-Ocln at Ser^490^, p-FAK at Ser^722^ and p-FAK at Tyr^576^ was dramatically decreased. Immunohistochemistry further indicated that phosphorylated levels of the two proteins were attenuated by the DSP and Ocln antibodies (Fig. 6A–C; Supplementary Fig. 5), indicating that DSP regulates phosphorylation of Ocln and FAK proteins via DSP-Ocln signaling.

### DSP induces dental cell differentiation and mineralization

**Figure 7:**
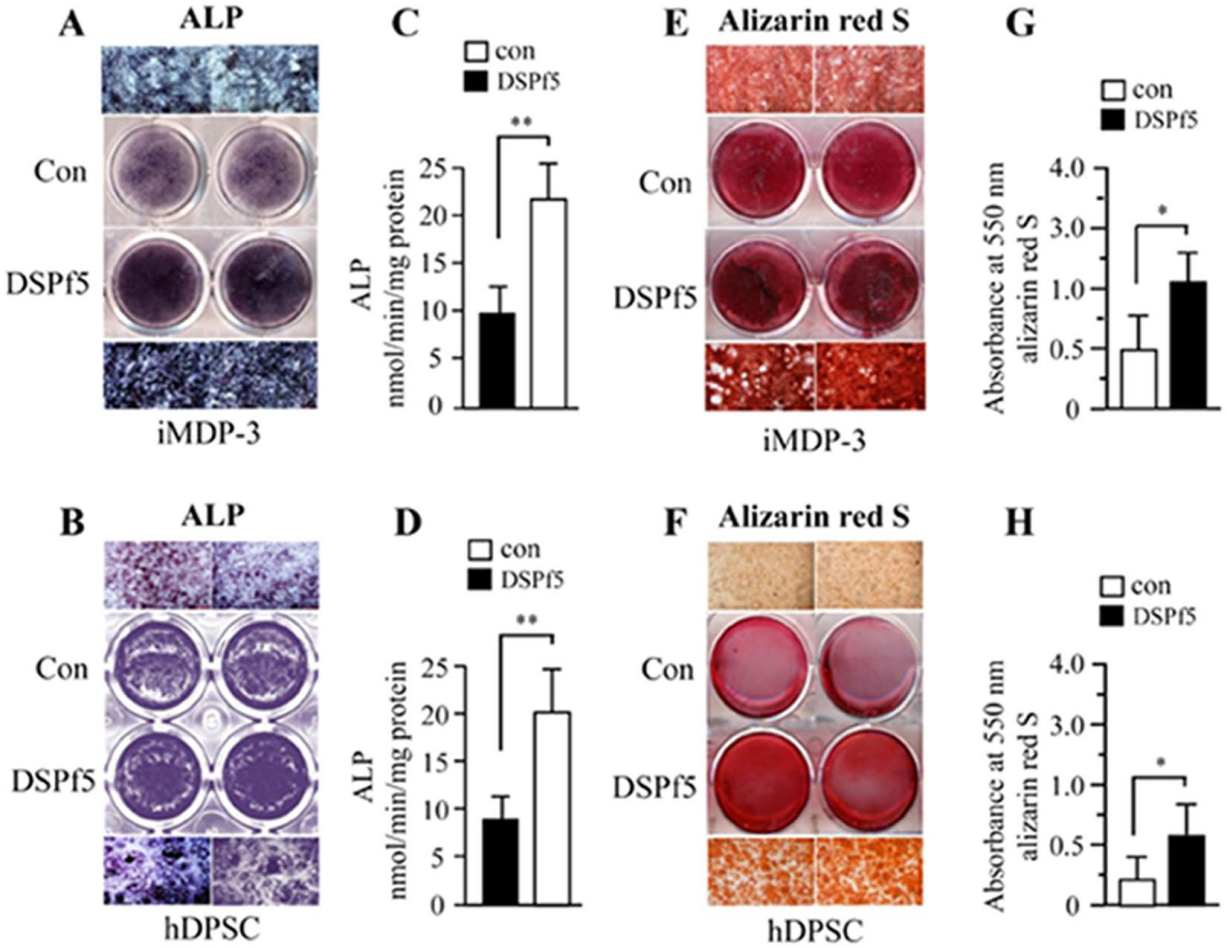
Effect of DSP domain on dental cell differentiation and mineralization. Mouse dental papilla mesenchymal (iMDP-3) (**A**) and human dental pulp stem (hDPSC) cells **(B)** were treated with or without 10 μg/ml of DSPf5 in calcifying medium for 7 days. ALP activity was analyzed using *in situ* ALP staining (**A**, **B**). (**C,D**) Quantitative ALP activity of the cell lysates was assayed using ρ-nitrophenyl phosphate as a substrate. Protein concentration was determined using the BCA protein assay reagent as described in “Materials and methods”. There were significantly different between the DSPf5 treated groups and DSPf5 untreated groups. For cell biomineralization, iMDP-3 (**E**) and hDPSC cells (**F**) were maintained in the same condition for 14 days. The cells were fixed and stained for Alizarin red S dye. (**G,H**) The amount of calcium deposition was quantified by destaining with 10% cetylpyridinium chloride in 10 mM sodium phosphate at room temperature. Mineralization deposits were determined and data represent mean ± S.D. (n = 3). Con, control. *p < 0.05; **p < 0.01.

As DSP is abundant in dentin ECM during odontoblast differentiation, we further addressed the possibility that Ocln-FAK signaling could be involved in controlling functional activities of dental cell differentiation and mineralization. To directly assess DSP effect on dental cell differentiation, we firstly examined differentiation of human dental pulp stem cells and mouse dental papilla mesenchymal cells in cultured systems. ALP assay revealed that DSPf5 has high effect on human dental pulp stem cell and mouse dental papilla mesenchymal cell differentiation compared to the control group (Fig. 7A–D). Alizarin red S assay also demonstrated that DSPf5 stimulates more calcium deposition of the two dental cells after 14 days of culture than that of the control groups (Fig. 7E–H). The cell differentiation and mineralization induced by DSPf5 protein was attenuated by the DSP and Ocln antibodies, whereas IgG as control had no effect on the cell differentiation and mineralization stimulated by DSPf5 (Supplementary Fig. 6). Furthermore, gain- and loss-*Ocln* gene in the mouse dental papilla mesenchymal cells up- and down-regulated the cell differentiation and mineralization (Supplementary Fig. 7). The data indicate that DSP promotes the dental cell differentiation and mineralization partially through the Ocln signaling.

**Figure 8:**
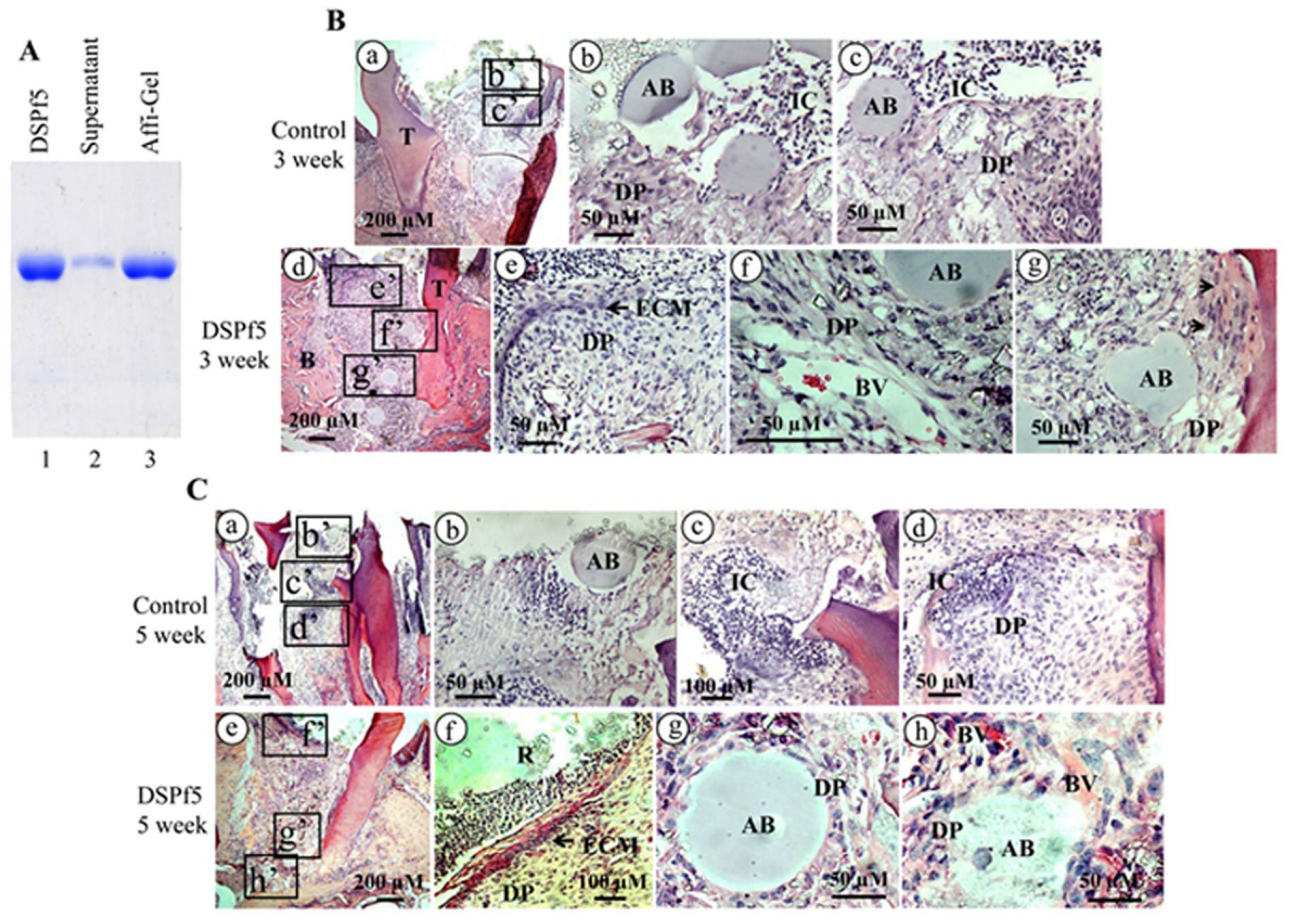
Effect of DSP domain on dental cell differentiation *in vivo*. (**A**) Lane 1, DSPf5 protein only; lane 2, DSPf5 was mixed with Affi-Gel Blue Gel Beads at room temperature for overnight. The mixture was centrifuged and supernatant was loaded onto a 7% SDS-PAGE gel; lane 3, the pellet was added to 1 x SDS-loading buffer and heated. The released DSPf5 from the compound was loaded onto a 7% SDS-PAGE gel. (**B**) The complex of DSPf5 coated to agarose beads was implanted into mouse dental pulp chambers. Compared to the control group after 3 week operation (**B**a–c), in the DSPf5-treated group, DSPf5 was able to induce the dental pulp cell proliferation and differentiation. Dental pulp cells in the DSPf5-treated group secrete ECM (arrow) at the top “artificial hole” between resin and dental pulp. Blood vessels (BV) proliferate and migrate near agarose beads (AB). Arrowheads show that dental pulp cells differentiate and secrete ECM. b, c and e–g are enlarged from the boxes in the b’, c’ and e’–g’. In DSPf5-treated group after 5 week operation (**C**), ECM secreted by dental pulp cells forms a layer covering “the artificial hole” between resin and dental pup cells (**C**e, f). Dental pulp cells surround the agarose beads (AB) and AB was resorbed and dental pulp cells and blood vessels invade into AB (**C**g, h). A number of inflammatory cells were decreased compared to the control group. b–d and f–h are enlarged from the boxes in the b’–d’ and f’–h”. B, alveolar bone; BV, blood vessels; DP, dental pulp cells; ECM, extracellular matrix; IC, inflammatory cells; R, resin; T, teeth.

To further assess whether this DSP peptide modulate endogenous dental mesenchymal cell differentiation and biomineralization in an *in vivo* context, we mixed the peptide with Affi-Gel Blue Gel. Data showed that DSPf5 highly binds to the Affi-Gel Blue Beads, forming a DSP-bead compound and this DSPf5 peptide is also easily released from the compound (Fig. 8A). Then, the compound was implanted into mouse dental pulp chambers. Tissue morphology after 1-, 3-, and 5-week surgery was observe by histological chemistry, and H&E stained tooth tissue sections revealed that there were not significant differences of tooth morphology between the control and DSPf5 treated groups after 1 week surgery (Supplementary Fig. 8a–f). However, after 3 week treatment of DSPf5, DSPf5 was able to induce the dental pulp mesenchymal cell proliferation and differentiation. Dental pulp mesenchymal cells in the DSP-treated group secrete dental ECM at the top “artificial hole” between the resin and dental pulp chamber (Fig. 8Ba-g). More interestingly, in the DSP-treated group, there were many blood vessels and less inflammatory cells around the agarose beads and dental pulp cells. Also, the agarose beads were resorbed as well as dental pulp cells and blood vessels were invaded into the agarose beads. Similar to the 3-week DSP treatment group, in the 5-week DSP induced groups, ECM covered major space at the wound area and less inflammatory cells were seen in the dental pulp cavity. Many blood vessels were apparent around the agarose beads (Fig. 8Ca–h). However, formation of reparative dentin was not seen in the DSP treated groups, the mechanism of the DSP domain in the reparative dentin formation needs to be further investigated in the future. These findings indicate that the DSP domain induces differentiation of dental pulp mesenchymal cells into odontoblast-like cells and differentiating cells are capable of synthesizing and secreting ECM.

## Discussion

DSP is highly expressed in dentin ECM during odontoblast differentiation and dentin formation. Its mutations are associated with dentin hereditary diseases^19–21, 44^. Previous studies showed that DSP and its fragments derived from DSP regulate intracellular signal transductions^9, 16, 26^. Unlike other SILING members, DSP domain does not contain RGD^8, 45^. Therefore, mechanisms of DSP in regulation of intracellular signaling during dentinogenesis have not well been understood. Recently, our and other laboratories found that DSP is processed by MMP20 and MMP9 into the given fragments^11, 15^ and the middle domain of DSP^aa 183–219^ binds to integrin β6, forming a complex. This complex phosphorylated transcription factors, Smad1/5/8, through p38 and Erk1/2 protein kinases and up-regulated expression of DSPP and DMP1 genes as well as induced dental mesenchymal cell attachment, differentiation and mineralization^9^. In this study, we used DSP as bait to screen protein library from mouse odontoblast-like cells and identified that several cell membrane proteins were bound by DSP including Ocln (Table 1). Previous studies showed that Ocln interacts with several cellular molecules and activates intracellular signal transductions^22, 29–31^. Ploss *et al*. identified that Hepatitis C virus (HCV) enter human hepatic cells through HCV glycoproteins (HCVpp) and OCLN is an essential entry factor. They furthermore found that the specific determinants of OCLN’s HCV entry factor functions are entirely contained within the OCLN extracellular loop 2^31^. As DSP is able to bind to Ocln *in vitro* and *in vivo*, we further studied which domain of DSP interacts with Ocln and found that the COOH-DSP domain^aa 363–458^ is a ligand and binds to the extracellular loop 2 (OclnL2) of Ocln. This DSP domain regulates intracellular signaling through a direct matrix-cell interaction. Immunohistochemistry showed that expression of DSP and Ocln is stage-specific in odontoblasts during dentinogenesis.

Ocln is an integral membrane protein expressed in epithelial and other cells and was originally predicted to confer barrier properties to the TJs^27–29^. During dentinogenesis, adjacent odontoblasts are tightly attached together and freeze-fracture studies showed that TJs between odontoblasts appear as short rows of fused particles during early mantle dentin mineralization and become more complex, eventually forming networks of fused particles in advanced mineralization^46–49^. The transport of calcium ions through odontoblasts is mediated by specific cell organelles and cell membrane domains^47, 48, 50–53^. TJs between odontoblasts are mainly composed of transmembrane proteins including claudin and Ocln as well as cytoplasmic proteins [such as zona occludens-1 (ZO-1), ZO-2 and ZO-3, FAK]^48, 49, 54^. Expression of the TJ-associated proteins was clearly detected in odontoblasts, however, temporal and spatial distributions of these proteins such as Ocln in differentiating odontoblasts are different^48, 49, 55–58^. The cause of the differences is still uncertain, and it may be due to the animals used and the methods for specimen preparation as well as antibody concentrations in each study^59^. Lee *et al.* observed that Ocln and ZO-1 proteins are strongly expressed in odontoblasts at PNs from 7 to 18 examined in the wild type mouse teeth whereas expression of Ocln and ZO-1 is hardly detected in odontoblasts of Nfic-deficient mice at the same ages. Nfic-deficient teeth failed to differentiate into normal odontoblasts and these aberrant odontoblasts were round and dissociated and lost their polarity. Electron microscopy of aberrant odontoblasts exhibited no intercellular junctional complex between them^58^. Lungrw *et al.* described that MRPC-11 cells derived from rat dental pulp cell line exhibit an odontoblast-like phenotype and express DSPP and Ocln. TJ complex was seen in the intercellular spaces of these cells and the transcellular Ca^2+^ flux was inhibited by nifedipine, giving evidence for an active intracellular Ca^2+^ transport through voltage-gated channels^60^. In this study, we observed that expression of DSPP/DSP and Ocln was not detectable in tooth tissues at embryonic days 13.5 (Fig. 2Ab,c). With odontoblast cytodifferentiation, both of these proteins were expressed in odontoblasts at PNs from 1 to 15 examined (Fig. 2Ag,h,l,m,q,r). Our results are in agreement with previous studies by other groups^5, 55–61^.

Ocln mutations in humans cause band-like brain calcification, polymicrogyria and advanced chronic renal disease^15, 33–35^. *Ocln* deficient mice developed deafness with dislocalization of tricellulin in cochlea^36^. Tricellulin is a recently identified constituent of TJs, and is the first marker of the tricellular tight junction (tTJ)^62^. Also, *Ocln* knock-out mice showed calcification in the brain, testicular atrophy, loss of cytoplasmic granules in striated duct cells of the salivary gland, and thinning of the compact bone, but in *Ocln* knockout-mice, morphology of the TJs does not appeared to be affected and barrier function of epithelium is normal^37^ and cells originating from *Ocln*-deficient embryonic stem cells have well-developed networks of tight-junction strands^38^, suggesting that the functions of Ocln are more complicated than previously supposed. These contradictory data led us to ask if Ocln contributes to a regulatory function in intracellular signal transductions. Our current study demonstrated that a novel role of Ocln acts as a receptor interacting with DSP for modulating intracellular functions via DSP-Ocln-FAK axis. It was found that several ECMs and molecules interacting with either extracellular loop 1 or 2 of Ocln regulate intracellular signaling through phosphorylation/dephosphorylation of Ocln^28–31, 63^. In the present study, we found that DSP^aa 363–458^ binds to the extracellular loop 2^aa 194–241^ of Ocln. Both of the DSP^aa 363–458^ and OclnL2^aa 194–241^ are high homologous across species lines. In human, the mutation of DSP at codon 45 (c.133C > T, p.Q45X) introduces a premature terminal signal and would results in a truncated protein without the COOH-terminal DSP domain^21, 25, 64, 65^. As the mouse COOH-terminal DSP domain^aa 363–458^ is highly homologous to the human COOH-terminal DSP domain^aa^ 374–469^66^ and this domain regulates dental mesenchymal cell lineages and mineralization through Ocln-FAK signal. It is assured that the lack of the domain of the human DSP in these cases (p.Q45X) may be relevant to DGI.

Evidence for Ocln phosphorylation has previously been described^28–30^. However, the mechanisms by which these modifications through DSP influence intracellular signaling during dentinogenesis have not been elucidated. We demonstrated that the DSP peptide phosphorylates Ocln at Ser^490^ in mouse dental papilla mesenchymal cells and the Ocln phosphorylation at Ser^490^ induced by DSPf5 was blocked by DSP and Ocln antibodies, implying Ocln phosphorylation at Ser^490^ through DSP signaling. Previously, it was found that vessel endothelial growth factor (VEGF) induces Ocln phosphorylation at Ser^490^ through activating protein kinase C (PKC) β and regulation of vascular permeability^63^. We observed that besides induction of Ocln phosphorylation, DSP also activated FAK phosphorylation at Ser^722^ and Tyr^576^ in mouse dental papilla mesenchymal cells. Maximal induction of Ocln and FAK phosphorylation was at 1- and 2-h of DSPf5 treatment. FAK is ubiquitously expressed in a variety of cells. Previous study showed that FAK is structurally associated with Ocln, but not other tight junction proteins, JAM-A and claudin-11 in Sertoli cells. FAK knock-down resulted in a significant loss of Ocln-ZO-1 interaction and interruption of the TJs^67^. Our studies demonstrated that similar to coexpression of DSP and Ocln, expression of both Ocln and FAK is stage-specific in odontoblasts during tooth development and in mouse dental papilla mesenchymal cells as well as Ocln physically interacts with FAK (Fig. 4). Expression of Ocln and FAK in odontoblastic cells during tooth formation was also observed by other groups^24, 58, 60^. Thus, coexpression of the three of DSP, Ocln and FAK exists in odontoblasts during tooth development. FAK bridges the cytoskeleton on the inside of cells with components of the ECM on the outside of cells via the cell surface receptors such as integrin and others^68, 69^. FAK protein contains many phosphorylated sites and phosphorylation events occurring within FAK influence numerous processes including mitogenic signaling, cell migration, proliferation and differentiation. Its activity is modulated by phosphorylation, either in a positive or negative fashion^70, 71^. FAK phosphorylation could be induced by adhesion of cell surface receptors such as integrin to ECM and by a variety of other extracellular factors^72^. Our study demonstrated that FAK phosphorylation at Ser^722^ and Tyr^576^ was blocked by either DSP or Ocln antibody. These findings suggest that FAK is a substrate of Ocln. However, the mechanism how Ocln phosphorylates FAK at Ser^722^ and Tyr^576^ remains unknown and whether the DSP affects binding affinity and structure of Ocln and FAK needs to be further investigated. In fact, recent studies have found that there are multiple potential Tyr, Ser and Thr phosphorylation sites in FAK mapped by mass spectrometry^73^.

**Figure 9:**
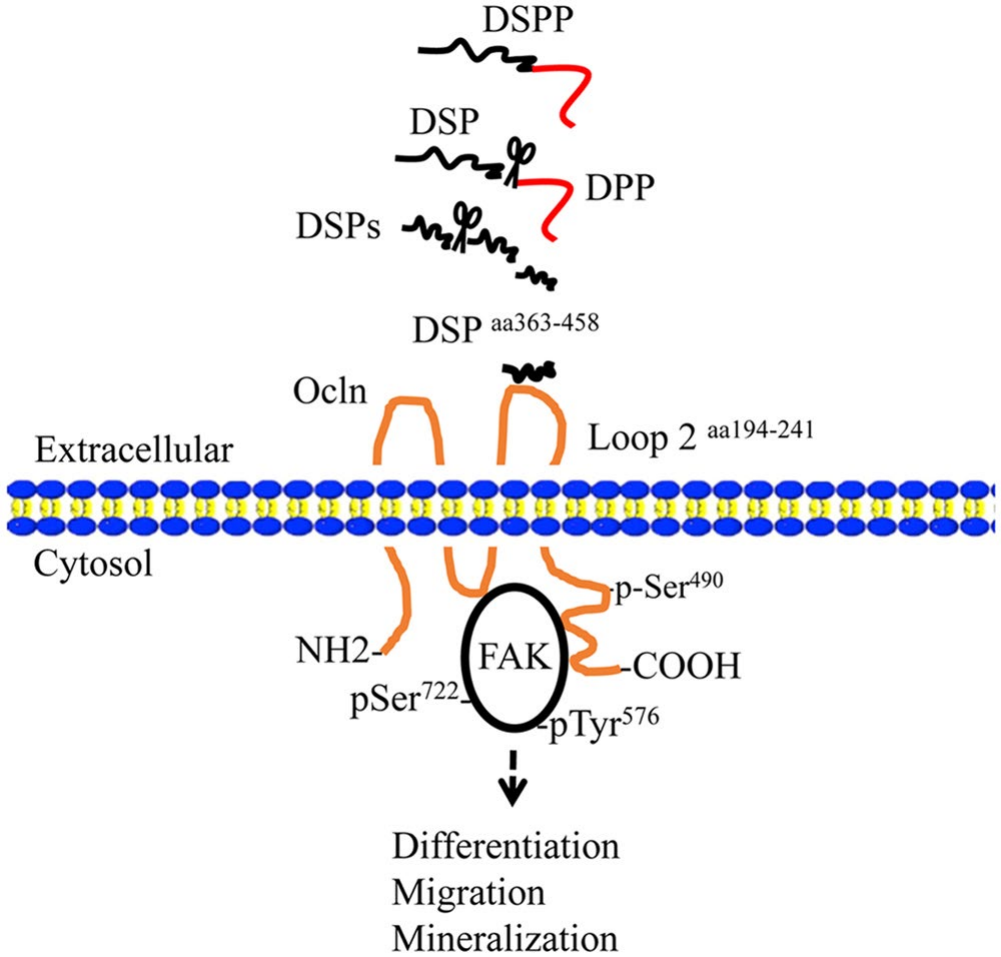
DSP domain regulates dental mesenchymal cell differentiation through occludin-FAK signaling. The hypothetical model depicts that the DSP^aa 363–458^ acts as a ligand and interacts with the extracellular loop 2 of Ocln^aa 194–241^, activating Ocln phosphorylation at Ser^490^. Furthermore, the DSP-Ocln complex activates FAK phosphorylation at Ser^722^ and Tyr^576^ and then induces dental mesenchymal cell differentiation and mineralization.

In this study, we found that DSP domain^aa 363–458^ interacts with the extracellular loop 2 of Ocln^aa 194–241^. The DSP domain induced phosphorylation of Ocln at Ser^490^ and FAK at Ser^722^ and Tyr^576^. The phosphorylation of Ocln and FAK proteins could be blocked by the anti-DSP and anti-Ocln antibodies, respectively. Immunohistochemistry analysis showed that co-expression of DSP, Ocln and FAK proteins are present in mouse odontoblasts and dental mesenchymal cells. Furthermore, *in vivo* study revealed that Ocln binds to FAK, indicating that the DSP domain regulates the intracellular activities through the Ocln-FAK signaling pathway. For the functional study, we observed that the DSP peptide^aa 363–458^ is sufficient to induce differentiation and mineralization of human dental pulp stem cells and mouse dental papilla mesenchymal cells in the cultured system. Effect of DSP on dental papilla mesenchymal cell differentiation and mineralization could be blocked by the DSP and Ocln antibodies and gain- and loss-*Ocln* gene in these cells was able to up- and down-regulate the cell differentiation and mineralization. More importantly, when this peptide was implanted into mouse dental pulp chambers, this DSP peptide was able to induce endogenous dental pulp cell proliferation, differentiation. The differentiating dental pulp cells were capable of synthesizing and secreting dental ECM and migrating to the region between resin and dental pulp chamber as well as new synthesized dentin ECM covered the wound area. Also, in DSP-treated groups, more blood vessels were seen surrounding dental pulp cells and agarose beads. Less inflammatory cells were observed in dental pulp chambers compared to the control groups. Although the formation of dentin extracellular matrix between the wound area and dental pulp chamber in the DSP treated groups in 3- and 5-week surgery was seen, the formation of the reparative dentin in both the two groups was not observed. The phenomenon remains to be further studied. In the present study, these findings suggest that this DSP peptide could be a potentially interesting therapeutic molecule by locally regenerating dentin-pulp complex. However, the detail mechanisms by which DSP domain mediates dental cell proliferation, differentiation, migration and blood vessel regeneration through Ocln-FAK signaling transduction needs to be further investigated in the future. Figure 9 depicts a model by which DSP domain^aa 363–458^ regulates dental cell differentiation and mineralization via DSP-Ocln-FAK signaling based on this report.

## Materials and Methods

### Antibodies and Reagents

A rabbit polyclonal anti-mouse DSP antibody recognizes residues between ILe18 and Lys371, (M-300), #sc-33587; goat polyclonal anti-mouse DSP (M-20), #sc-18328; rabbit polyclonal anti-mouse p-FAK (Tyr-397), #sc-11765-R; rabbit polyclonal anti-mouse p-FAK (Tyr-576), #sc-16563-R; mouse monoclonal anti-mouse p-FAK (Ser-722), #sc-374668; rabbit polyclonal anti-mouse FAK, #sc-557; rabbit polyclonal anti-Akt, #sc-5298; rabbit polyclonal anti-mouse p-Akt1/2/3 (Ser-473), #sc-7985-R; goat polyclonal actin, #sc-1616 antibodies, mouse *Ocln* shRNA plasmid #sc-36118 were purchased from Santa Cruz Biotechnology Inc. (Santa Cruz, CA, USA). A polyclonal anti-DSP-COOH antibody was produced in rabbit using the oligopeptide with the sequence of KRNSPKQGESDKPQGTAE (mouse DSP residues 401–418; Alpha Diagnostic International, San Antonio, TX, USA, #2603). Monoclonal anti-Myc-, #13–2500; anti-GFP, #33-2600; and anti-Flag, #MA1-91878 antibodies were purchased from Thermo Fischer Scientific (Waltham, MA, USA). A monoclonal occludin antibody was obtained from Life Technologies (Carlsbad, CA, USA, #33-150). A rabbit antibody against occludin phosphorylated at Ser-490 was kindly provided by Dr. Antonetti (Department of Cellular and Molecular Physiology and Ophthalmology, Penn State College of Medicine, Pennsylvania, USA). Normal mouse immunoglobulin G (IgG) (Vector Laboratories, Burlingame, CA, USA, #I-2000) was employed as a negative control.

### Animals and tissue preparation

All experimental procedures involving the use of animals were approved by the University of Texas Health Science Center at San Antonio (UTHSCSA, approval number: IACUC-50012X), TX, USA. ICR mice were purchased from Harlan-Laboratory Animals Inc. (Indianapolis, IN. USA). The tissue sections were prepared as previously described in detail^74^. Mice with litters of embryonic day (E) 13.5 and postnatal days (PN) 1, 5, 10 and 15 were sacrificed. Mouse tissues were dissected and fixed in 4% paraformaldehyde overnight. After demineralization in 10% EDTA, samples were dehydrated in increasing concentrations of ethanol, embedded in paraffin. Serial sections (4 μM) were prepared for immunohistochemistry analysis. All experimental methods were conducted in accordance with relevant guidelines.

### Cell culture

Mouse dental papilla mesenchymal cells^75^ and human dental pulp stem cells^76^ were grown in alpha minimal essential medium (a-MEM) (Life Technologies, USA) with 10% fetal calf serum (FCS, Atlanta Biologicals Inc., Norcross, GA, USA), 50 µg/ml ascorbic acid, 10 mM sodium β-glycerophosphate, 100 units/ml penicillin/streptomycin (Sigma–Aldrich, St. Louis, MO, USA) at 37 °C under 5% CO_2_. Human embryonic kidney (HEK-293) cells were purchased from American Tissue Collection Center (ATCC, Manassas, VA, USA**;** #CRL-1573) and were maintained in Dulbecco’s modified eagle medium (DMEM) with 10% FCS, 100 units/ml penicillin/streptomycin at 37 °C under 5% CO_2_.

### Generation of recombinant dentin sialoprotein and occludin

The expression and purification of a recombinant DSP were performed according to previously described protocols^16^. Full-length and different fragments of mouse DSP cDNA were amplified by PCR using a full-length mouse DSPP cDNA as a temple with primers shown in Supplementary Table 2 adding EcoRI sites at both ends for directional ligation into the expression vector pGEX-6P3 with EcoRI sites (Amersham Biosciences, Piscataway, NJ, USA) and named DSP, DSP-N, DSP-C, DSPf1, DSPf2, DSPf3, DSPf4 and DSPf5 (Fig. 1G). Mouse *Ocln* plasmid (pBluescript SK-*Ocln*) was kindly provided by Dr. Mikio Furuse (Department of Physiology and Cell Biology, Kobe University Graduate School of Medicine, Kobe, Hyogo, Japan). Full-length and different fragments of *Ocln* cDNA including the whole length and extracellular loops 1 (OclnL1) and 2 (OclnL2) of *Ocln* gene were amplified by PCR using a full-length mouse *Ocln* cDNA as a template. Primers used for generation of *Ocln* DNA constructs were shown in Supplementary Table 2. PCR products were subcloned into pGEX-6P3 vector, respectively (Amersham Biosciences, USA). After confirming the right sequence, the resulting plasmid was transformed into *Escherichia coli* BL21. The protein expression and purification were performed according to the manufacturer’s instruction (Amersham Biosciences, USA). Briefly, the fusion protein was induced by the addition of 1 mM of isopropyl b-D-thiogalacto pyranoside (IPTG) at 37 °C for 5 h. The recombinant DSP and Ocln proteins were then purified using a GSTrap 4B protein purification system (Amersham Biosciences, USA, #28-4017-45). The purified recombinant proteins were analyzed by sodium dodecyl sulfate–polyacrylamide gel electrophoresis (SDS–PAGE), followed by Coomassie brilliant blue staining and Western blotting analysis using GST antibody (1:2,000, Amersham Biosciences, USA, #27-45-77-01). For *in vivo* study, DSP and *Ocln* genes were subcloned into CMV expression vector tagged with either Myc or Flag (Sigma-Aldrich, USA, #pp2393, #pp2395). Mammalian mouse FAK with GFP expression plasmid was obtained from Addgene (Cambridge, MA, USA, #50515).

### Glutathione fusion protein (GST) pull down assay

GST pull down assay was according to the manufacture’s instruction (Amersham Biosciences, USA). In brief, GST-DSP fusion protein was incubated with 1 ml of cell lysis from mouse odontoblast-like cells (MO-6G3)^6^ in the cold lysis buffer containing 50 mM Tris-HCl pH 7.4, 150 mM NaCl, Triton X100 (Sigma-Aldrich, USA) overnight at 4 °C. After the reaction, the 50 μl of a 50% slurry of glutathione agarose beads (Amersham Biosciences, USA) were added for further incubation for 2 h at 4 °C. The samples were centrifuged at 12,000 g for 2 min at 4 °C and the supernatant was removed. After extensive washes containing 10 mM Tris-HCl, pH 8.0, 150 mM NaCl, 0.025% sodium azide, the DSP binding proteins were eluted by 20 mM reduced glutathione in 50 mM Tris-Cl, pH 8.0. The eluted samples (10 μg) were mixed with 2x SDS-PAGE gel loading buffer after boiling and run onto 7% SDS-PAGE gels and were transferred to Trans-Blot membranes (Bio-Rad Laboratories Inc., Hercules, CA, USA). The membrane was blocked with 5% non-fat milk in TBST buffer (10 mM Tris-HCl, pH 7.5, 100 mM NaCl, 0.1% Tween-20) for 60 min at room temperature. After blocking, the membranes were incubated overnight at 4 °C with the respective primary antibodies (1:500-1,000 dilutions) shown in Table 1 and Supplementary Table 1. The membranes were washed with 1x TBST and incubated with diluted horseradish peroxidase (HRP)-conjugated secondary antibodies (1:5,000, Pierce, Rockford, IL, USA) for 1 h at room temperature. After three washes, the membranes were detected using an enhanced chemiluminescence (ECL) kit (Millipore, Bedford, MA, USA).

### Co-immunoprecipitation

Co-immunoprecipitation assay was performed according to the manufacture’s instruction (Active Motif, Carlsbad, CA, USA, #54002). Briefly, HEK 293 cells from a 100-mm Petri dish (70–90% subconfluent) were transfected with 20 μg of Flag and c-Myc plasmids containing DSP and *Ocln* genes using the Lipofectamine 2000 (Life Technology, USA). After 48 h transfection, the cells were lysed in 3 mL of lysis buffer containing 50 mM Tris-HCl, pH 7.4, 150 mM NaCl, 1 mM EDTA, 1% Triton X-100 and proteinase inhibitor cocktail (Sigma-Aldrich, USA) and mixed with a vortex mixer for 1 h at 4 °C. Insoluble materials were removed by centrifugation at 12,000 g for 10 min at 4 °C. Lysates containing 5 mg of proteins in 1 mL were precleared by incubating with 40 μl of anti-FLAG or anti-Myc Affinity Gel (Sigma-Aldrich, USA, #A4596 and #IP0020) at 4 °C on a rotator overnight. Immunoprecipitates were washed three times with 1 x TBST and heated for 5 min at 95 °C in 20 µl 2x  SDS-PAGE sample buffer (62.5 mM Tris HCL, pH 6.8. with 2%SDS, 10% glycerol, 0.002% bromophenol blue). The bound materials were resolved on 7% SDS-PAGE gels for analysis by Western blotting with anti-Ocln (1:1,000, Life Technologies, USA), or anti-Myc (1:1,500; Thermo Fischer Scientific, USA) or anti-DSP (1:1,000, Santa Cruz Biotechnology Inc., USA) antibody. *In vitro* immunoprecipitation of DSP and Ocln fusion proteins was performed according to the manufacture’s protocol (Amersham Biosciences, USA). In brief, 10 μg of GST-DSP and - Ocln fusion proteins were pre-cleared with 10% protein G-strepharose in binding buffer. The samples were then incubated with 10 μg of either primary DSP (Santa Cruz Biotechnology Inc., USA) or Ocln (Life Technologies, USA) antibody, followed by immunoprecipitation with 20 μl protein G-strepharose (Amersham Biosciences, USA). Immunoprecipitations were washed extensively with lysis buffer (50 mM Tris-HCl, pH 7.4, 150 mM NaCl, 1 mM EDTA, 1% Triton X-100) and boiled for 5 min. The supernatants were separated by 7% SDS-PAGE gels, transferred to nitrocellulose membranes, immunoblotted with primary DSP (1:1,000, Santa Cruz Biotechnology Inc., USA) or Ocln (1:1,500, Life Technologies, USA) antibody at 4 °C overnight. The membranes were then washed with 1 × TBST for three times and incubated with diluted horseradish peroxidase (HRP)-conjugated secondary antibodies (1:5,000, Pierce, USA) for 1 h at room temperature. After three washes, the membranes were detected using an enhanced chemiluminescence (ECL) kit (Millipore, USA).

### Biotinylation of dentin sialoprotein and substrate binding assay

For probing protein–protein interactions *in vitro*, recombinant DSP^aa 363–458^ (DSPf5) and Ocln loop2^aa 194–241^ (OclnL2) proteins at concentrations of 300 mg/ml was dialyzed against 0.1 M NaHCO_3_ and then reacted with 100 mg/ml Sulfo-NHS (N-hydroxysuccinimido)-LC (long-chain)-biotin according to the manufacture’s instruction (Pierce, USA) for 20 min at room temperature, followed by 2 h at 4 °C, respectively. Free biotin was removed by dialysis against 50 mM Tris-HCl and 150 mM NaCl, pH 7.4. To characterize the relative DSPf5 interaction with OclnL2, substrate binding assay was performed as described earlier^77^. Briefly, 96-microwell plates were coated with 1 μg/per well of OclnL2 and bovine serum albumin (BSA) as control overnight at 4 °C and non-specific binding sites were blocked with 1% BSA (Sigma-Aldrich, USA) at room temperature for 1 h. After through rinses with 1x PBS, the biotinylated DSPf5 ranged from 0–18 fold molar excesses in PBS with 1% BSA was added into the plates and then incubated at room temperature for different time periods. Bound DSPf5 was reacted with AP-conjugated streptavidin diluted 1:10,000 in 1x PBS for different time points at room temperature using 1 mg/ml PNPP (ƿ-nitrophenyl phosphate disodium) as substrate (Pierce, USA) and quantified at 405 nm (Opsys MR, Dynex, Chantilly, VA, USA). BSA was used as a control group and the binding of DSPf5 to OclnL2 was expressed as relative binding activity compared to the control group. At the same way, binding of the biotinylated OclnL2 to the unlabeled DSPf5 was performed. All experiments were performed from three independent experiments in triplicate.

### Protein sequences and data analyses

A database search was performed at the National Center for Biotechnology Information website (http://www.ncbi.nlm.nih.gov/blast) using the BLAST program. The DSPP and *Ocln* nucleotides and derived amino acids were aligned with those from different species using the Gene Runner software program (http://www.generunner). Accession numbers of mRNA and protein sequences of DSPP and Ocln were shown in Supplementary Fig. 2.

### Alkaline phosphatase (ALP) and mineralization assays

For detection of ALP activity, cultures of the human dental pulp stem and mouse dental papilla mesenchymal cells at 6-well palates with 10^4^ cells per well were treated with or without DSPf5 (10 μg) induction in calcifying medium (α-MEM supplemented with 3% FBS, penicillin (100 U/ml) and streptomycin (100 μg/ml), 50 μg/ml ascorbic acid, 10 nM dexamethasone and 10 mM sodium β-glycerophosphate) at 37 °C for 7 days and fixed with 70% ethanol for 5 min and washed with 1 x PBS. *In situ* ALP staining was performed according to the manufacture’s instruction (Bio-Rad Laboratories Inc., USA). Quantitative ALP activity of the cell lysates was assayed using ρ-nitrophenyl phosphate as a substrate. Protein concentration was determined using the BCA protein assay reagent (Pierce, USA). The enzyme activity was expressed as nanomoles of ρ-nitrophenol produced per min per mg of protein. For mineralization assay, calcium deposition was determined according to the manufacture’s recommendation protocol (Sigma–Aldrich, USA). In brief, these cells were seeded into 6-well plates and treated with or without 10 μg of DSPf5 in calcifying medium at 37 °C for 14 days. The cells were fixed in 10% formaldehyde neutral buffer at room temperature for 15 min. The monolayers were then washed twice with excess ddH_2_O prior to addition of 1 mL of 40 mM alizarin red S dye (pH 4.1) per well (Sigma–Aldrich, USA). The plates were incubated at room temperature for 20 min with gentle shaking. After aspiration of the unincorporated dye, the wells were washed four times with ddH_2_O while shaking for 5 min. Stained monolayers were visualized by phase microscopy using a Nikon Eclipse TE2000S inverted microscope with a filter by means of a digital cooled camera connected to a PC computer and analyzed with NIS-Elements 3.2 software (Melville, NY, USA). The amount of calcium deposition was quantified by de-staining with 1 ml of 10% cetylpyridinium chloride (Sigma–Aldrich, USA) in 10 mM sodium phosphate at room temperature for 1 h. The dye was then removed and 200 μL aliquots were transferred to a 96-well plate prior to reading at 550 nm using a microplate reader (Opsys MR, USA). Experiments were performed in triplicate and repeated in three cultures (n = 3).

### RNA preparation and reverse transcription-polymerase chain reaction (RT-PCR)

Total RNA was extracted from the mouse dental papilla mesenchymal cells using RNA STAT-60 kit (Tel-Test, Inc. Friendswood, TX, USA, #cs-110), treated with DNase I (Promega, Madison, WI, USA, #M6101), and purified with the RNeasy Mini Kit (Qiagen Inc., Valencia, CA, USA, #74104). RNA concentration was determined at an optical density of OD_260_. The RNA was transcribed into cDNA by SuperScript II reverse transcriptase according to the manufacture’s instruction (Life Technologies, USA, #18-080-044). qPCR was performed in a 20 μl reaction containing 2 ρmol of specific primers as follows: DSPP forward, 5′-AACTCTGTGGCTGTGCCTCT-3′; reverse, 5′-TATTGACTCGGAGCCATTCC-3′; *Ocln* forward, 5′-CATAATGGGAGTGAACCCGAC-3′, reverse, 5′-TATAGCCTCCTGGGGATC-3′. The PCR reaction was first denatured at 95 °C for 5 min, and then carried out at 95 °C for 60 s, at 55 °C–60 °C for 60 s and at 72 °C for 60 s for 30 cycles and with a final 10 min extension at 72 °C in T100TM Thermal Cycler (Bio-Rad Laboratories Inc., USA, #1861096). Five μl of PCR products were analyzed by 1.5% agarose gels with ethidium bromide staining.

### Immunohistochemistry

In order to examine the expression of DSP, Ocln and FAK during tooth development and dental papilla mesenchymal cells, the fluorescent immunohistochemistry was performed according to previous descriptions^74^. In brief, tissue sections were dewaxed in xylene for 15 min for 3 times, hydrated in gradually decreasing ethanol concentrations for 2 min each and immersed in ddH_2_O for 5 min for three times. Antigen retrieval was performed by treating the sections with 0.1% (w/v) trypsin (Sigma-Aldrich, USA) at room temperature for 15 min. The sections were successively pretreated with 0.3% hydrogen peroxide for 30 min, blocked with 10% normal donkey serum (Sigma–Aldrich, USA). For double labeling, two primary antibodies were incubated simultaneously overnight at 4 °C at the following dilutions: rabbit anti-FAK (1:100; Santa Cruz Biotechnology Inc., USA, #sc-557), goat anti-DSP (1:100; Santa Cruz Biotechnology Inc., USA, #sc-18328), mouse anti-Ocln (1:100; Life Technologies, USA, #33-150), which were recognized by donkey anti-rabbit, anti-mouse and anti-goat IgG (H + L) antibodies conjugated with Alexa Fluo 488 and Alex Fluo 568 (Molecular Probes, Eugene, Ore., USA, #SA5-10166, #SA5-10168, #A110556, #A21206). After being washed with PBS, the sections were incubated with the secondary antibody (1:500) for 90 min at room temperature and then rinsed in PBS. For nuclear staining, the tissue sections were incubated with 1:5,000 dilution of Hoechst (Sigma-Aldrich, USA, #23491-45-4) for 5 min at room temperature. After being washed, the tissue sections were mounted in Vectashield mounting medium (Vector Laboratories, Burlingame, CA, USA). As a negative control, the primary antibody was replaced by 10% of mouse IgG (Vector Laboratories, USA, #I-2000). For cell double labeling, cells were fixed in cold acetone and methanol (1:1), permeabilized in 0.2% Triton X-100 at ice for 30 min and blocked with 10% donkey serum for 30 min at room temperature and two primary antibodies recognized by the donkey secondary antibody were incubated simultaneously overnight at 4 °C at the following dilutions: rabbit anti-FAK (1:100; Santa Cruz Biotechnology Inc., USA, #sc-557), goat anti-DSP (1:100; Santa Cruz Biotechnology Inc., USA, #sc-18328), mouse anti-Ocln (1:100; Life Technologies, USA, #33-150). After being washed, the cells were incubated with the secondary antibody conjugated with Alexa Fluo 486 green and Alexa Fluo 568 red (1:500; Molecular Probes, USA, #A21206, #SA5-10168) for 1 h at room temperature. For nuclear staining, the cells were incubated with 1:5,000 dilution of Hoechst (Sigma-Aldrich, USA, #23491-45-4) for 3 min at room temperature. After being washed, the cells were mounted in Vectashield mounting medium (Vector Laboratories, USA). As a negative control, the primary antibody was replaced by 10% mouse IgG (Vector Laboratories, USA, #I-2000). Images of Alexa Fluo 488 and Alex Fluo 568 staining of the various proteins were captured by a Nikon Eclipse TE2000S microscope with a filter by means of a digital cooled camera connected to a PC computer and analyzed with NIS-Elements 3.2 software. Images of nuclear staining with Hoechst were obtained via filter UV-2E/C, C86826. For each experiment, all slides were simultaneously processed for a specific antibody, so that homogeneity in the staining procedure was ensured between samples.

### Effect of dentin sialoprotein on phosphorylation of occludin and FAK

Mouse dental papilla mesenchymal cells were treated with or without recombinant DSPf5 (2-24 μg/ml) in DMEM medium with 1% antibiotics at 37 °C in 5% CO_2_ atmosphere at given time periods. The cells were then washed with PBS and lysed with RIPA buffer (1 x PBS, 1% Nonidet P-40, 0.5% sodium deoxycholate, 0.1% SDS, 10 mg/ml phenylmethylsulfonyl fluoride, 30 μl/ml aprotinin, 100 mM sodium orthovanadate; Santa Cruz Biotechnology Inc., USA). To characterize effect of DSPf5 on Ocln partners, Western blot assay was performed as described earlier^16^. Whole cell lysates were resolved by 7% SDS-PAGE gels and transferred to Trans-Blot membranes (Bio-Rad Laboratories, Inc., USA). The membrane was blocked with 5% non-fat milk in 1 x TBST buffer for 60 min at room temperature. After washing, the membranes were incubated with primary antibodies specific to Ocln (1:1,000, Life Technologies, USA, #33-150), p-Ocln-ser490 (1:500), FAK (1:1,000; Santa Cruz Biotechnology Inc., USA, #sc-557), p-FAK-ser722 (1:800, Santa Cruz Biotechnology Inc., USA, #sc-374668), p-FAK-tyr576 (1:1,000, Santa Cruz Biotechnology Inc., USA, #sc-16563-R), p-FAK-tyr-397 (1:1,000, Santa Cruz Biotechnology Inc., USA, #sc-11765-R), Akt (1:1,000, Santa Cruz Biotechnology Inc., USA, #sc-5298), p-Akt-ser473 (1:1,000, Santa Cruz Biotechnology Inc., USA, #sc-7985-R), and β-actin (1:1,500, Santa Cruz Biotechnology Inc., USA, #sc-1616) for overnight at 4 °C, respectively. The membranes were washed with 1 × TBST and incubated with diluted horseradish peroxidase (HRP)-conjugated secondary antibodies (1:5,000-10,000, Pierce, USA) for 1 h at room temperature. After three washes, the membranes were detected using an enhanced chemiluminescence (ECL) kit (Millipore, USA). For block of effect of DSP on Ocln and FAK phosphorylation, the cells were treated with either DSPf5 or DSPf5 plus DSP antibody (8 or 16 μg/ml) or Ocln antibody (8 or 16 μg/ml) in DMEM medium with 1% antibiotics at 37 °C in 5% CO_2_ atmosphere for 1 h. The cells were harvested and phosphorylation of Ocln and FAK were carried out by Western blot assay.

### Reparative dentin regeneration

Affi-Gel Blue (cross-linked agarose beads with covalently coupled Cibacron Blue F3-GA) was purchased from Bio-Rad laboratories Inc. and mixed with the DSP peptide (2 mg/ml). The mixture was centrifuged and pellet used for implantation. The experimental procedures were approved by the animal research committee of UTHSCSA. Twenty-five adult mice (C57BL/6) aged 1.5 months were anaesthetized by sodium pentobarbital (Sigma-Aldrich, USA). The first molars on the maxilla were cleaned by sterilized instruments. Exposed pulped cavities were prepared by ¼ diamond cylindrical burs with sterile saline cooling. The exposed pulped cavity on the left first molars was filled onto Affi-Gel blue gel as control and Affi-Gel blue gel with the DSP peptide on the right first molars. The cavities over the implanted materials were filled with Vitremer Glass Ionomer (GI) Core Build-Up/Restorative (Sku# 3303PEDO, 3 M ESPE, Dental Products, St. Paul, NM, USA) and sealed by light curing. Sealant well done in each animal was observed carefully. The animals were euthanatized at 1, 3, and 5 weeks after surgery. Tissue specimen were fixed in 4% paraformaldehyde at 4 °C overnight and embedded in paraffin wax after demineralization with 10% EDTA. The paraffin sections (5 μm in thickness) were morphologically examined after staining with hematoxylin and eosin (HE) (Agilent Technologies, USA).

### Statistical analysis

Quantitative data were presented as means S.D. from three independent experiments and compared with the results of one-way ANOVA using GraphPad Prism 5 (GraphPad Software, Inc. La Jolla, CA, USA). The differences between groups were statistically significant at *p < 0.05 and **p < 0.01.

## Electronic supplementary material

Dentin sialoprotein facilitates dental mesenchymal cell differentiation and dentin formation
