# Supplementary material for: Dentin sialoprotein facilitates dental mesenchymal cell differentiation and dentin formation
Source: Sci Rep. 2017 Mar 22;7:300. doi: 10.1038/s41598-017-00339-w (PMC5428264; doi:10.1038/s41598-017-00339-w)
Supplement: Supplementary file 1 — Dentin sialoprotein facilitates dental mesenchymal cell differentiation and dentin formation [file 41598_2017_339_MOESM1_ESM.pdf]

Title: Dentin sialoprotein facilitates dental mesenchymal cell differentiation and dentin formation

Wentong Li,[1, 2] Lei Chen,[3] Zhuo Chen,[1] Lian Wu,[1] Junsheng Feng,[1] Feng Wang,[1] Lisa Shoff,[1] Xin Li[1], Kevin J. Donly,[1] Mary MacDougall,[4] and Shuo Chen[1]*

[1]Department of Developmental Dentistry, the University of Texas Health Science Center at San Antonio, San Antonio, Texas, 78229-3700, United States

[2]Department of Pathology, Weifang Medical University, Weifang, Shandong Province, 261053, China.

[3]Department of Surgery, the First Affiliated Hospital, Fujian Medical University, Fuzhou, Fujian, 350108, China

[4]Department of Oral/Maxillofacial Surgery, University of Alabama at Birmingham School of Dentistry, Birmingham, Alabama, 35294-0007, United States

Supplementary Materials

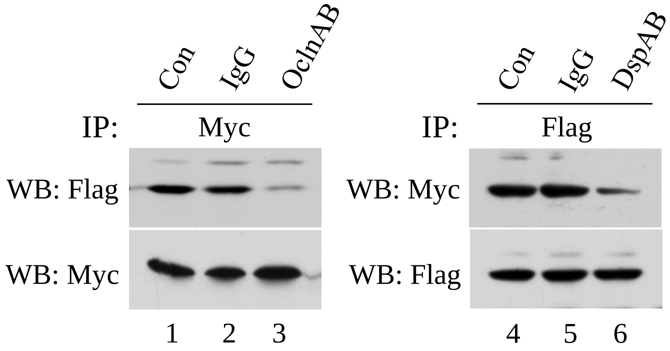

Supplementary Figure 1. Block of interaction of DSP and Ocln by DSP and Ocln antibodies. Five μg of Myc-DSP and Flag-Ocln mammalian expression vectors were transfected into HEK-293 cells. After 48h transfection, the proteins were harvested and isolated proteins were added with or without either 5 μg/ml of DSP or Ocln antibody or IgG as control in the binding buffer, respectively. The protein-protein interaction between DSP and Ocln was immunoprecipitated using Myc or Flag antibody. Protein-protein interaction was detected by Western blotting using anti-Myc or anti-Ocln antibody. Lanes 1 and 4 as control (Con); lanes 2 and 5, the isolated proteins were added to 5 μg/ml of IgG; lanes 3 and 6, the harvested proteins were added to 5 μg/ml of anti-DSP or anti-Ocln antibody, respectively. DSP-Ocln Interaction was blocked by DSP and Ocln antibodies. IP, immunoprecipitation; OclnAB, Ocln antibody; DspAB, Dsp antibody.

A

```
       363
        .           .         .           .         .          .         .
Mouse  -GIETE-GPNKGNKSIITKESGKLS-GSK-DSNG-HQGVELDKRNSPK-QGESDKPQGTAEKSAAHSN-LGHSRIG
Rat    -.L...-.SST....S.........-...-....H..-M.........-.........A....DT.-.NM......
Human  K...I-K..SS..R-N....V..GNE..E.K.-Q..--MI.G.G.V-.T...VVNIE.PGQ..EPG-.KV...NT.
        .         .         .   458      GenBank Accession No.
       SSSNSDGHDSYEFDDESMQGDDPKSSD       NM_010080
       ...........D...........N...       NM_012790
       .D.....Y...D...K.......N...       NM_014208
```

B

	Extracellular loop 2 amino acid sequences	GenBank Accession No.
	194 241	
Mus	GVNPTAQASGSMYGSQIYMICNQFYTPGGTGLYVDQYLYHYCVVDPQE	U49185.1
Rattus	..................T..S..........................	NP_112619.2
Canis	...........L.S....AM.....ASTA...................	NP_001003195.1
Bos	...........L.S....AL.......AA...................	NP_001075902.1
Sus	...........L.S....AL.......AA...................	NP_001157119.1
Frog	....V......AFYT..VS......S.VQ..VF.N.........E...	NP_001081943.1
Homo	.......S...L......AL.......AA...................	AAB00195.1
Gorilla	.......S...L......AL.......AA...................	XP_00458803.1
Orcinus	...........L.S....AL.......AAA.V................	XP_004270832.1
Zebrafish	T.Y.M..T...VQFN.V.SM.AAYQN.QMS.AF.N.............	AAH49304.1

Supplementary Figure 2. Alignment of amino acid sequences in the COOH-terminal regions of DSP and extracellular loop 2 of occludin across species. (A) Alignment of amino acid sequences in the COOH-terminal regions of DSP $^{aa\ 363\text{-}458}$ between the mouse, rat and human. The top strand depicts the mouse DSP amino acid sequences from 363 to 458 relative to the translational start site of DSPP protein taken as No. 1. The next two strands depict the rat and human sequence, respectively. Based identical to the mouse is indicated by dots, mismatches by letters, and gaps by dashes. Homologous amino acid sequences between the mouse and rat, and mouse and human show 85% and 58% in this regions. **(B)** Comparison of the extracellular loops of the mouse Ocln with that of other species lines. The top strand depicts the extracellular loop 2 of mouse Ocln from amino acids 194 to 241. Letters show differences of amino acids from that of the mouse.

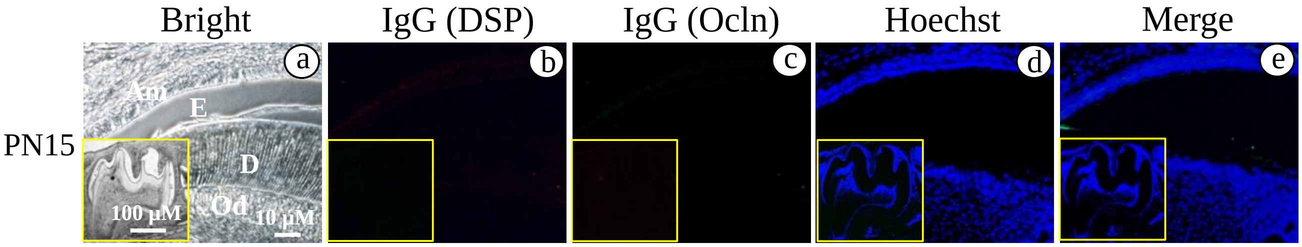

Supplementary Figure 3. Expression of dentin sialoprotein and occludin in developing mouse teeth. As control, the antibodies of DSP and Ocln in the tissue section at postnatal (PN) day 15 were replaced with normal IgG (**b** for DSP) and (**c** for Ocln). After being washed, the slides were incubated with the secondary antibody conjugated with Alexa Fluo 486 green (**b**) and Alexa Fluo 568 red (**c**) for 1 h at RT. Cellular nuclei were stained with Hoechst (**d**). **a** shows bright image. Image was merged (**e**). **a-e** are higher magnifications of the yellow boxes, respectively.

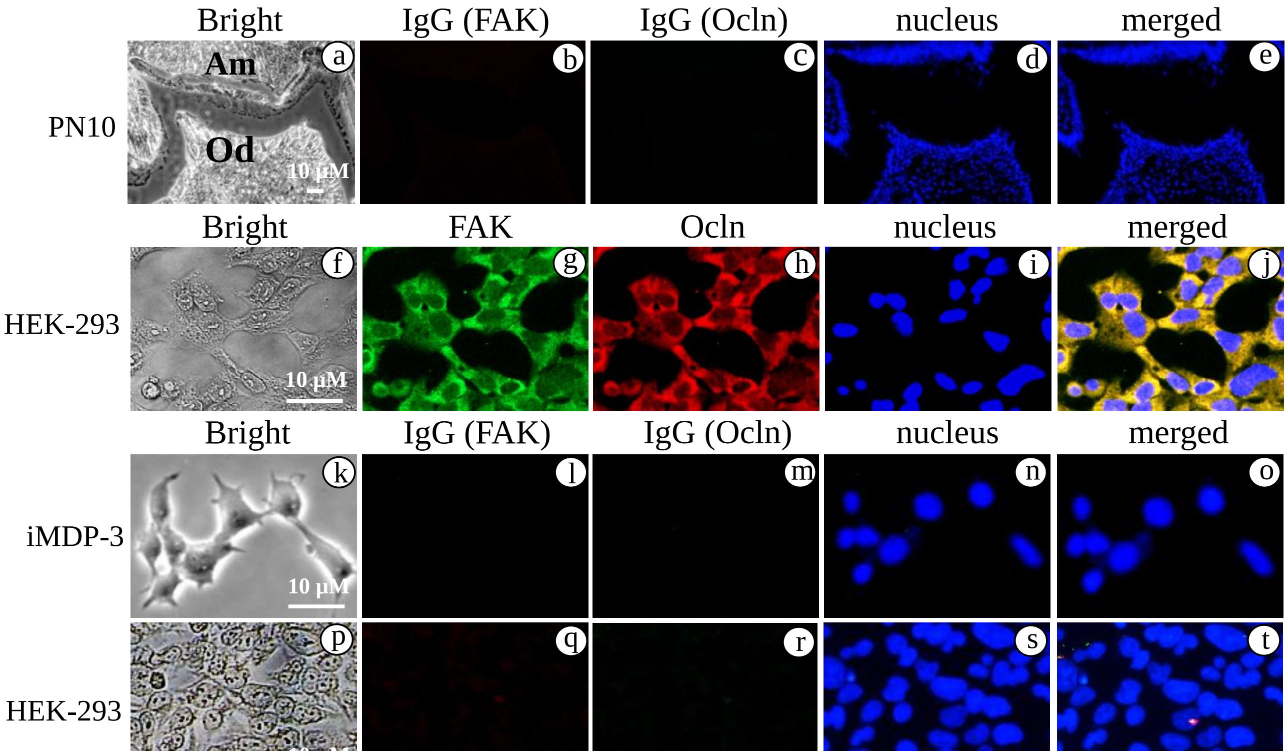

Supplementary Figure 4. Expression of FAK and occludin in developing mouse teeth. As negative control, the antibodies of FAK and Ocln in the tissue section at PN 10 were replaced with normal IgG (**b**

for FAK) and (**c** for Ocln), followed by incubation with the secondary antibody conjugated with Alexa Fluo 486 green (**b**) and Alexa Fluo 568 red (**c**) for 1 h at RT. Then, the slide was stained with Hoechst and image observed under the fluorescent microscope. The tissue section was photographed under a light microscope using a Nikon camera (**a**). Nuclei were stained with Hoechst (**d**). Image was merged (**e**). For positive control, coexpression of FAK with green color (**g**) and Ocln with red color (**h**) was detected in HEK-293 cells and shown in the merged image (**j**), respectively. Hoechst (blue) was use for nuclear staining (**i**). For negative control, the antibodies of FAK (**l, q**) and Ocln (**m, r**) were replaced with mouse IgG in iMDP-3 and HEK-293 cells. After being washed, the cells were incubated with the secondary antibody conjugated with Alexa Fluo 486 green and Alexa Fluo 568 red, followed by Hoechst staining. The images were observed under a fluorescent microscope with a Nikon camera. **k** and **p** were bright images. Nuclei were stained with Hoechst staining (**n, s**). Images were merged (**o, t**).

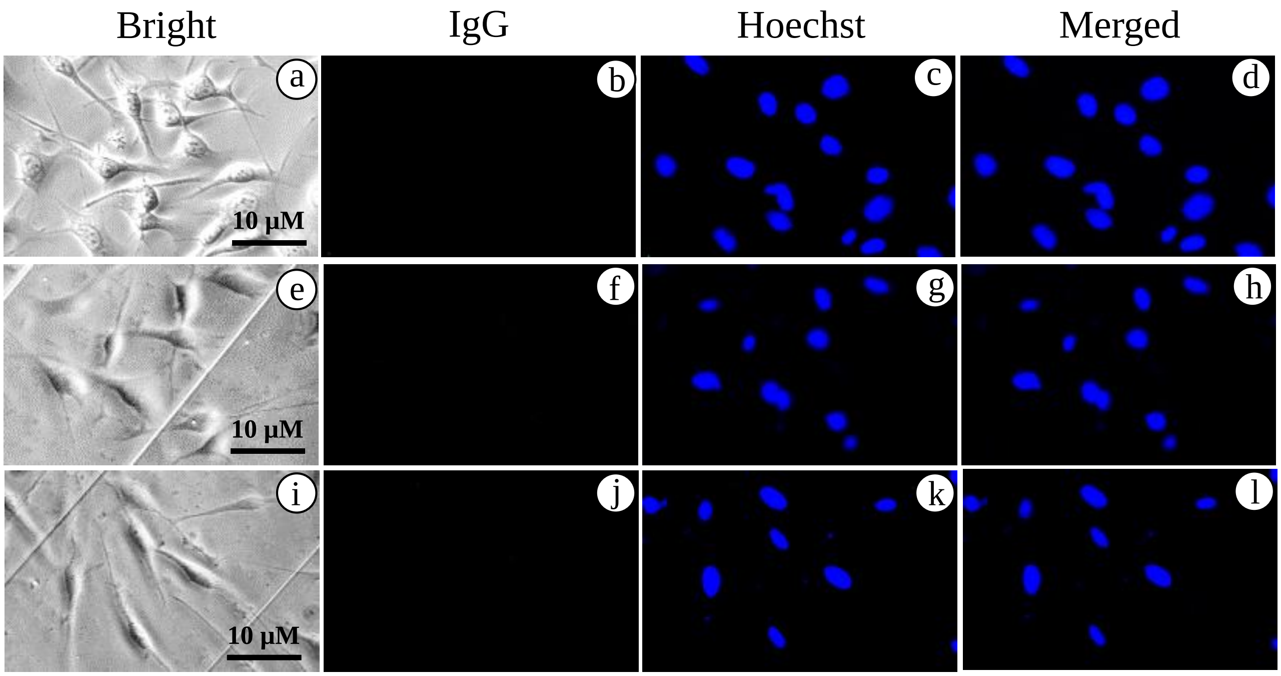

Supplementary Figure 5. Effect of DSPf5 on occludin and FAK phosphorylation is blocked by DSP and occludin antibodies. As control, the antibodies of p-Ocln-Ser^{490} (**b**), p-FAK-Ser^{722} (**f**) and p-FAK-Tyr^{576} (**j**) were replaced by normal Ig G. After being washed, the cells were incubated with the secondary antibody conjugated with Alexa Fluo 486 green, then followed by Hoechst staining. The slides were observed under the fluorescent microscope with a Nikon camera. **a, e** and **i** show bright images. Nuclei were stained with Hoechst dye (**a, g, k**). Images were merged (**d, h, l**).

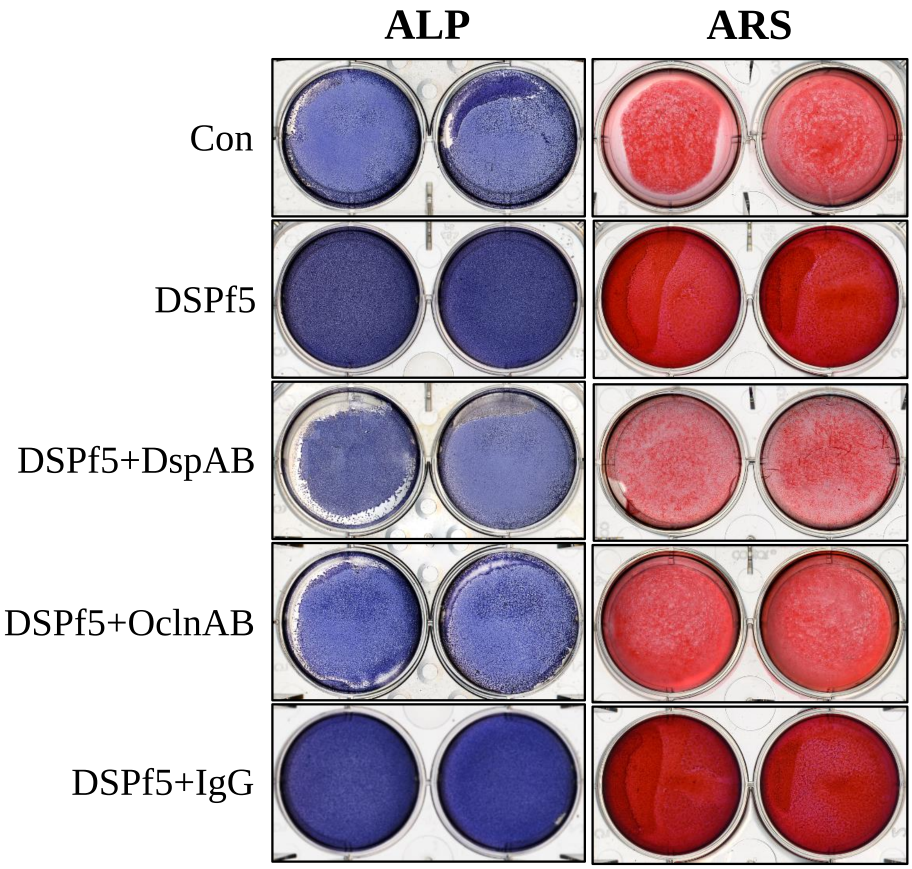

Supplementary Figure 6. Block of DSP effect on cell differentiation and mineralization by DSP and Ocln antibodies. The mouse dental papilla mesenchymal (iMDP) cells were treated with or without DSPf5 (8 μg/ml), or DSPf5 (8 μg/ml) plus 8 μg/ml of DSP antibody or DSPf5 (8 μg/ml) plus 8 μg/ml of Ocln antibody or DSPf5 (8 μg/ml) plus 8 μg/ml of IgG as control for 7 and 14 days, respectively. ALP activity was analyzed using in situ ALP staining. The cellular mineralization was stained for Alizarin red S (ARS) dye. The cell differentiation and mineralization induced by DSPf5 were attenuated by the DSP and Ocln antibodies.

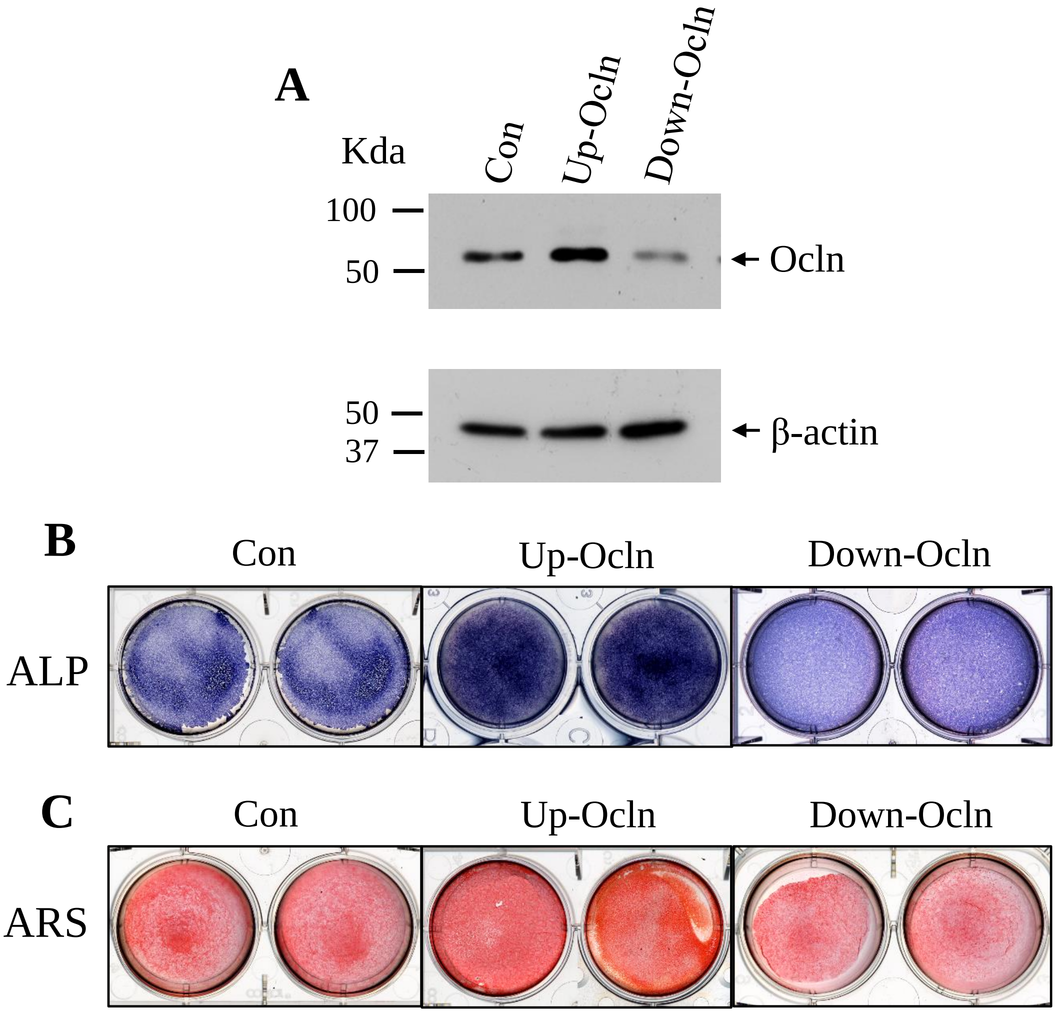

Supplementary Figure 7. Ocln effect on dental cell differentiation and mineralization. (**A**) Mouse dental papilla mesenchymal (iMDP-3) cells were transfected without or with either *Ocln* or *Ocln* shRNA expression plasmid. After 48 hours, the proteins were harvested and Western blot assay was performed using anti-Ocln antibody. Con, control; Up-Ocln, Ocln gene overexpression; Down-Ocln, Ocln shRNA vector. (**B-C**) iMDP-3 cells were transfected without or with either 5 µg of Ocln or Ocln shRNA expression plasmid and the cells were grown in calcifying medium for 5 and 10 days, respectively. At 5 day transfection, ALP activity was analyzed using in situ ALP staining (**B**). The cellular mineralization was stained for Alizarin red S (ARS) dye after 10 day transfection (**C**). The experiments were performed from three separate time points. kDa, protein ladder.

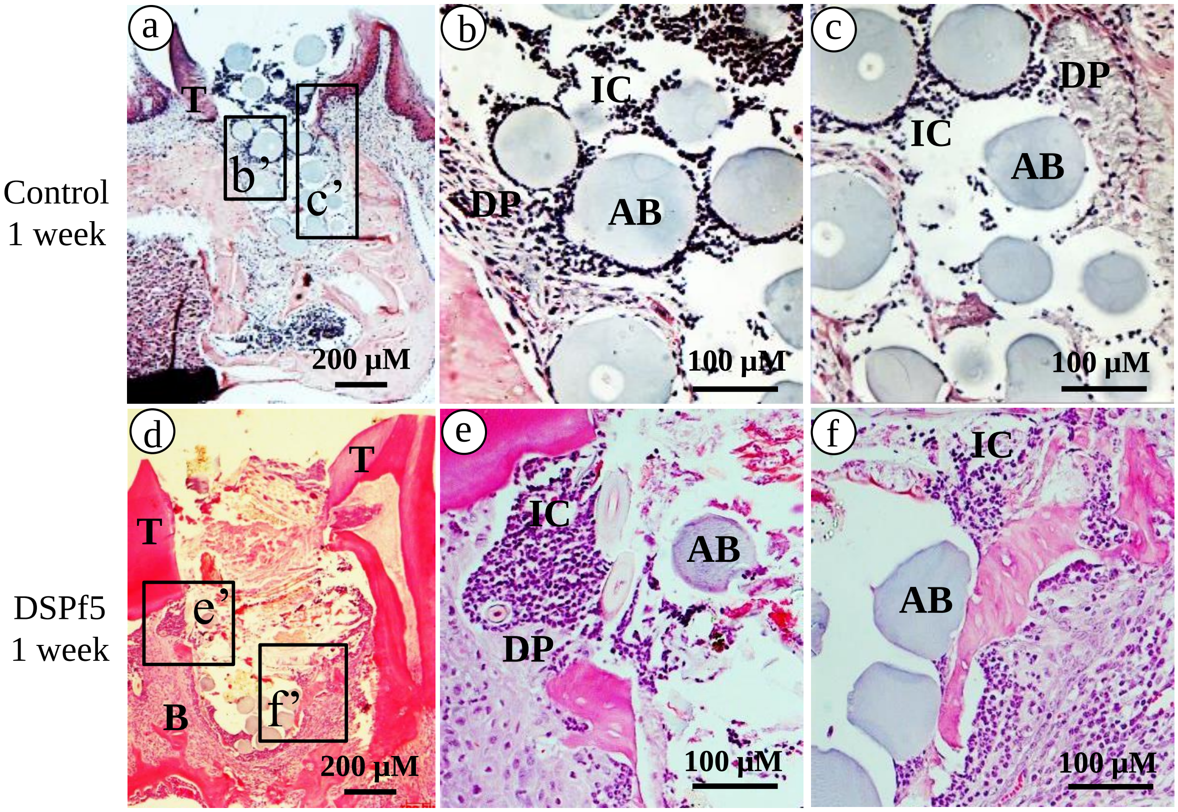

Supplementary Figure 8. Effect of DSP domain on dental cell differentiation in vivo. The complex of DSPf5 coated to agarose beads was implanted into mouse dental pulp chambers. In 1 week operation, there are no significant differences for dental pulp healing between the control (**a-c**) and DSPf5-treated groups (**d-f**). **b, c** and **e, f** are enlarged from the boxes in **b', c'** and **e', f'**.

Supplementary Table 1. Proteins Used for Binding Assay

Actin, Adenlyate kinases-2, A-kinase anchoring protein-150, Alkaline phosphatase (ALP), Amelogenin, Aquaporins-4,-9; ATF3, Bax, Bcl-2, Bcl-XL, Bone sialoprotein, Cell adhesion molecules (HCAM, ICAM-1,VCAM), Core binding factor (CBF-A, CBF-B, CBF-C), CBP, C/EBP-α, C/EBP-β, C/EBP-γ, CollIII, CollV, CREB, Decorn, Dlx3, Dlx5, EGF receptor (Neu-N), Fibronectin, Heat shock protein-27, HIF-1α, HIF-3α, Histone acetylase (GCN5, p300), hnRNP-K, Dentin matrix protein1, Enamelysin (MMP-20), EMSP1, Glutathione–S-transferase (GST), Insulin-like growth factor-I, -II; Integrin-α2, IntegrinαIIb, Integrinα5, IntegrinαV, IntegrinαVβ3, Integrinβ1, Integrinβ3, Integrinβ7, Integrinβ8, IL-1, IL-1R, c-Jun N-terminal kinase-1, Keratin, Laminin, Leukemia inhibitory factor, Myc, NF-κB, Nitric oxide-1, -2, -3; OPG, Osteonectin, Osteirx, Osteocalcin, Osteopotin, Peripherin, Protein kinase C, RANKL, Ribosomal S6 kinase-1, -2, -3; Runx2, E-selectin, L-selectin, P-selectin, SPARC, Synaptophysin, Syndecan-1,-2, -3, -4; T cell factor-4, Tenascin-C, Tissue inhibitor of metalloproteinases (TIMP-1, -2, -3, -4), Toll-like receptors (TLR-2, -3, -4), Tissue plasminogen activator (tPA), TRPA1, TGF β receptor associated binding protein, Transforming growth factor-β2, Transforming growth factor-β-RII, Tumor necrosis factor receptor, TNF-a, TNF-R1, TNF-R2, Tuftelin, Urokinase-type plasminogen activator (uPA), Vascular endothelial growth factor (VEGF), Vimentin, Vitronectin.

Supplementary Table 2. Primer sequences used for generation of DSP and Ocln constructs

Primers	Sequences
DSP_{1-89}-F	ATGAAAATGAAGATAATTATA
DSP_{1-89}-R	TATTGACTCGGAGCCATTCCC
DSP_{72-191}-F	CAGGTACTTAGCGAGGATGGTT
DSP_{72-191}-R	GCTATTCTTGATGCTAGCTTC
$DSP_{182-295}$-F	GAGCCTCCAGAAGCTAGCATC
$DSP_{182-295}$-R	TTCAGTACTAACTGA ACTCTG
$DSP_{263-371}$-F	GGAGATGGA AGGGAGAGCCAT
$DSP_{263-371}$-R	TTTGTTGGGACCTTCAGTTTC
$DSP_{369-463}$-F	CCCAACAAAGGCAACAAAAGTA
$DSP_{369-463}$-R	ACTTCCGTTAGATTCGTCGCTG
DSP_{1-213}-F	ATGAAAATGAAGATAATTATA
DSP_{1-213}-R	TTCTCTCTGAGGCGTCGTTTC
$DSP_{203-463}$-F	GTTGCTACACATGAAACGACG
$DSP_{203-463}$-R	ACTTCCGTTAGATTCGTCGCTG
DSP_{1-463}-F	ATGAAAATGAAGATAATTATA
DSP_{1-463}-R	ACTTCCGTTAGATTCGTCGCTG
OclnL-$_{85-133}$-F	TTCCACACTTGCTTGGGACAG
OclnL-$_{85-133}$-R	GAAGCCTTTGGCTGCTCT
OclnL-$_{192-243}$-F	CATAATGGGAGTGAACCCGAC
OclnL-$_{192-243}$-R	TATAGCCTCCTGGGGATCAA
Ocln-$_{1-521}$-F	ATGTCCGTGAGGCCTTTTGA
Ocln-$_{1-521}$-R	CTAAGGTTTCCGTCTGTCAT
